# Comprehensive review on patulin and *Alternaria* toxins in fruit and derived products

**DOI:** 10.3389/fpls.2023.1139757

**Published:** 2023-04-03

**Authors:** Syed Asim Shah Bacha, Yinping Li, Jiyun Nie, Guofeng Xu, Lingxi Han, Saqib Farooq

**Affiliations:** ^1^ Laboratory of Quality & Safety Risk Assessment for Fruit, Research Institute of Pomology, Chinese Academy of Agricultural Sciences, Xingcheng, Liaoning, China; ^2^ College of Horticulture, Qingdao Agricultural University/Laboratory of Quality & Safety Risk Assessment for Fruit (Qingdao), Ministry of Agriculture and Rural Affairs/National Technology Centre for Whole Process Quality Control of FSEN Horticultural Products (Qingdao)/Qingdao Key Lab of Modern Agriculture Quality and Safety Engineering, Qingdao, China

**Keywords:** patulin, *Alternaria*, detection method, fruits, biosynthesis pathways, management strategies

## Abstract

Mycotoxins are toxic secondary metabolites produced by certain fungi, which can contaminate various food commodities, including fruits and their derived products. Patulin and *Alternaria* toxins are among the most commonly encountered mycotoxins in fruit and their derived products. In this review, the sources, toxicity, and regulations related to these mycotoxins, as well as their detection and mitigation strategies are widely discussed. Patulin is a mycotoxin produced mainly by the fungal genera *Penicillium*, *Aspergillus*, and *Byssochlamys*. *Alternaria* toxins, produced by fungi in the *Alternaria* genus, are another common group of mycotoxins found in fruits and fruit products. The most prevalent *Alternaria* toxins are alternariol (AOH) and alternariol monomethyl ether (AME). These mycotoxins are of concern due to their potential negative effects on human health. Ingesting fruits contaminated with these mycotoxins can cause acute and chronic health problems. Detection of patulin and *Alternaria* toxins in fruit and their derived products can be challenging due to their low concentrations and the complexity of the food matrices. Common analytical methods, good agricultural practices, and contamination monitoring of these mycotoxins are important for safe consumption of fruits and derived products. And Future research will continue to explore new methods for detecting and managing these mycotoxins, with the ultimate goal of ensuring the safety and quality of fruits and derived product supply.

## Graphical Abstract

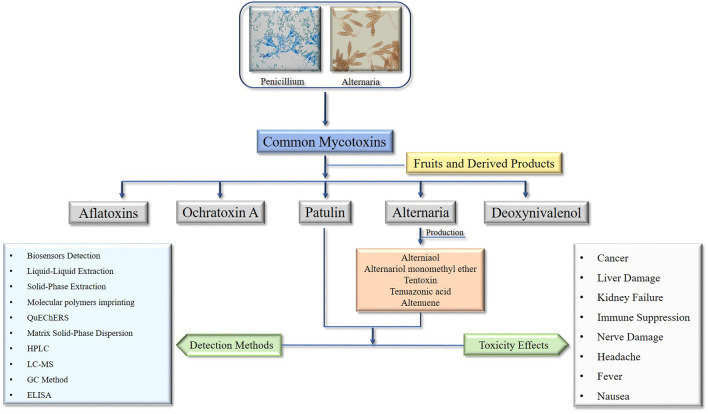

## 1 Introduction

Fruits are essential nutritional sources for humans and a staple of the human diet. Mycotoxins contamination is a significant cause of production loss and a threat to consumer health and safety (Xue et al., 2010; Zhou et al., 2014; Rizzo et al., 2021). Due to their structural stability and resistance to heat, mycotoxins are difficult to remove during food processing and can ultimately persist in food and food products (Barkai-Golan and Paster, 2008; Oliveira et al., 2014). In certain countries, mycotoxin contamination can affect up to 50% of agricultural products, while in general, approximately 25% of agricultural products are affected by these toxins (Adeyeye and Oyewole, 2016; Graybill and Bailey, 2016). As a result, numerous countries and international organizations have established regulatory limits for mycotoxins (Li et al., 2020b; Notardonato et al., 2021). Even at low concentrations, mycotoxins exhibit a wide range of biological activities, including teratogenic, mutagenic, carcinogenic, and cytotoxic effects (Shi et al., 2019). As reports of food safety issues continue to increase, concerns over food safety problems resulting from mycotoxin contamination have grown among the public worldwide (Li et al., 2020b). Fungi produce low molecular weight mycotoxins, and although over 300 mycotoxins have been reported, only a limited number pose a threat to the health of humans and animals (Vejdovszky et al., 2017; Kermany et al., 2018). The most common mycotoxins in fruits and their derived products are patulin produced by *penicillium* species, *Alternaria* mycotoxins produced by *Alternaria* species (Fernández-Cruz et al., 2010; Li et al., 2020b).

Patulin is a significant mycotoxin found in various fruits, predominantly produced by *P*. *expansum* during storage. Its presence in baby food, including homogenized and fruit juices, is a major concern. Moreover, the International Agency for Research on Cancer categorizes patulin as a Group 3 substance, which means it has the potential to cause cancer, but there is currently insufficient evidence to confirm its carcinogenicity (Alshannaq and Yu, 2017; Notardonato et al., 2021). Although it has received relatively less attention compared to other mycotoxigenic fungi, *Alternaria* mycotoxin is a significant source of mycotoxins and can cause stem and leaf spot diseases, as well as spoil fruits and kernels during postharvest stages. As a result of its high prevalence in various food commodities and the presence of its toxins in fruits and their derived products, there has been a surge in scientific research on this fungal genus in recent years (Patriarca et al., 2019). Due to its ability to grow at low temperatures, the *Alternaria* genus is primarily responsible for the spoilage of numerous fruits, and their products during long-distance transport and refrigerated storage (Liang et al., 2016).

*Alternaria* and patulin, two types of more vivacious mycotoxins, can be found in fruits and fruit-derived products. These mycotoxins can be harmful to human health, and therefore their generation, accumulation, biosynthesis, and detection methods are of great interest to food safety researchers and practitioners (Mahakham et al., 2016). The generation and accumulation of *Alternaria* and patulin mycotoxins in fruits and fruit-derived products are influenced by various factors, such as temperature, humidity, storage conditions, and the presence of other microorganisms. For instance, high humidity and warm temperatures are conducive to the growth of *Alternaria* and *Penicillium* fungi, which can lead to increased mycotoxin production (Gutarowska et al., 2015). The biosynthesis of *Alternaria* mycotoxins involves various enzymes, including polyketide synthases (PKSs), non-ribosomal peptide synthetases (NRPSs), and cytochrome P450 monooxygenases (CYPs) (Villena et al., 2020). The biosynthesis of patulin involves a complex pathway that includes several enzymatic reactions. Various methods are available for the detection of *Alternaria* and patulin mycotoxins in fruits and fruit-derived products, including: High-performance liquid chromatography (HPLC) that separates and quantifies mycotoxins based on their chemical properties, enzyme-linked immunosorbent assay (ELISA) that bind to specific mycotoxins and the presence of mycotoxins in a sample can be detected using colorimetric or fluorescence methods, mass spectrometry (MS) to identify and quantify mycotoxins based on their mass-to-charge ratio, and Biosensors that use biological components to detect mycotoxins in food products to detect specific mycotoxins or a range of mycotoxins (Freire and Sant’Ana, 2018). Subsequently, *Alternaria* and patulin mycotoxins are a concern in fruits and fruit-derived products due to their potential harmful effects on human health. Researchers and practitioners use various methods to detect and quantify these mycotoxins in food products. Understanding the factors that influence their generation and accumulation can help mitigate the risk of mycotoxin contamination.

In view of the great scientific importance of patulin and *Alternaria*, mycotoxins considered to be the most vibrant contaminants in fruits, this review is organized with the objectives to comprehensively evaluate the *Alternaria* and patulin mycotoxin generation in fruits and their derived products along with the accumulation, biosynthesis, and detection methods of these mycotoxins in fruit and derived products, to ensure food safety, improving quality control, and developing new products.

## 2 Mask and emerging mycotoxins

Mycotoxins are toxic compounds produced by fungi that grow on nuts, fruits, and their derived products. They can contaminate fruits and products and pose a health risk to humans and animals if ingested. Masked mycotoxins are a class of mycotoxins that are not readily detected using standard analytical methods. They are formed when a mycotoxin binds to other compounds in the food matrix, such as proteins or sugars, making it difficult to detect and quantify (Berthiller et al., 2013). However, these masked mycotoxins can be converted back into their active form in the body during digestion, potentially causing adverse health effects. Researchers are continuing to identify new mycotoxins and study their potential health effects (Grace et al., 2015). Emerging mycotoxins are newly discovered mycotoxins or those that have only recently been recognized as a potential health risk. They may not yet be regulated or monitored in food products, and their toxicity and prevalence are still being studied. Some examples of emerging mycotoxins include enniatins, beauvericin, and alternariol. Alternariol is a mycotoxin produced by several species of *Alternaria* fungi (Gruber-Dorninger et al., 2017). It has been found in a range of fruits and their products, and has been associated with genotoxic and immunotoxic effects (Gruber-Dorninger et al., 2017). One of the characteristics of *Alternaria* mycotoxins is that they can occur as “masked” mycotoxins. Masked mycotoxins are mycotoxin conjugates that are formed when mycotoxins bind to other molecules, such as sugars or amino acids, in the plant material. These conjugates are not usually detected by conventional mycotoxin analysis methods, as the mycotoxin is masked and not present in its free form (Freire and Sant’Ana, 2018). However, during digestion, the conjugate can be broken down, releasing the free mycotoxin. Masked *Alternaria* mycotoxins can pose a health risk, as they may not be detected by routine testing methods, and their potential toxicity is not well understood. The European Food Safety Authority (EFSA) has identified AOH and AME as potential masked mycotoxins of concern and has recommended further investigation into their occurrence and toxicity (Capriotti et al., 2010). In the case of patulin, it is a relatively stable compound and can be detected and quantified using standard analytical methods (Freire and Sant’Ana, 2018). While patulin is not widely considered an emerging mycotoxin, it is still a significant concern because of its potential health effects. Patulin has been associated with acute toxicity, including nausea, vomiting, and diarrhea, as well as long-term health effects such as immunotoxicity and genotoxicity (Barac, 2019b). However, it is important to note that patulin can be degraded during food processing or storage, which can result in the formation of other toxic compounds (Marin et al., 2013). Additionally, patulin can also interact with other compounds in food, which may affect its bioavailability and toxicity. Therefore, even though patulin is not considered a masked mycotoxin, it is still important to monitor and control its presence in food products (Arroyo-Manzanares et al., 2021).

Several methods are available for the detection of masked mycotoxins in food commodities. These include immunoassays, chromatographic methods, and mass spectrometry (Anfossi et al., 2016). Immunoassays are sensitive and relatively simple to perform, but they can produce false positive results. Chromatographic methods, such as high-performance liquid chromatography (HPLC), are commonly used for the detection of mycotoxins. These methods can separate the components of a sample and identify specific mycotoxins based on their retention time and spectral properties. Mass spectrometry is a powerful analytical technique used for the detection of mycotoxins. This method can provide high sensitivity, specificity, and selectivity, making it useful for the detection of masked mycotoxins (Singh and Mehta, 2020). Several studies have reported the presence of masked patulin and *Alternaria* mycotoxins in various food commodities. For instance, masked patulin was detected in apple juice, applesauce, and apple cider vinegar samples by using HPLC coupled with fluorescence detection (Li et al., 2020b). In another study, masked *Alternaria* mycotoxins were found in tomato sauce, chili powder, and paprika samples by using LC-MS/MS. The masking of mycotoxins in food commodities can make their detection challenging. Patulin and *Alternaria* mycotoxins are two types of mycotoxins that can be masked, and they pose significant health risks to consumers. Several methods, including immunoassays, chromatographic methods, and mass spectrometry, are available for the detection of masked mycotoxins. Careful monitoring of food commodities is essential to prevent the exposure of consumers to these harmful mycotoxins (Iqbal, 2021).

## 3 Patulin

Patulin is a synthesized mycotoxin produced by several species of the genera *Aspergillus* and *Penicillium*, and it has been identified in many vegetables, cereals, moldy fruits, and other foods. Patulin has a low molecular weight, composed of alpha-beta unsaturated gamma lactone, which can contaminate many different foods, especially fruit and their products (Ngolong Ngea et al., 2020). It can produce citrinin, ochratoxin A, patulin, penitrem A, and rubratoxin B. Patulin has been identified in tomatoes, other fruit crops, and several consumer products, including dehydrated (dried) products (Zheng et al., 2018; Biango-Daniels et al., 2019; Saleh and Goktepe, 2019). Fruits contain a lot of water and sugar, which increases patulin activity (Iqbal et al., 2018; Zhong et al., 2018; Saleh and Goktepe, 2019; Solairaj et al., 2020). Patulin was a vital fruit concentrate (Zheng et al., 2018) in many juices, compote mixtures, commercial apple-based beverages, and baby foods (Zheng et al., 2018). Patulin has drawn global attention because it exacerbates health risks as it has mutagenic, carcinogenic, neurotoxic, genotoxic, immunotoxic, and gastrointestinal effects on human and animal health. In the 1960s, patulin was used for treating common colds and nose infections because of its antiviral, antiprotozoal and antibacterial properties. Later, it was classified as a true mycotoxin because of its toxic effects on human and animal health (Piqué Benages et al., 2013; Zaied et al., 2013).

Patulin also appeared to be severely hazardous in the post-harvest life of fruits, starting from single grain to the contamination of whole fruit and ending up spoiling the entire stored fruits (Hussain et al., 2020). Because of the blue mould decay and the subsequent production of patulin, the profitability of fruit producers has been jeopardized, as has human health (Cummings et al., 2018). It is one of the hazardous mycotoxins in fruits and induces a series of acute symptoms, gastrointestinal disturbances, vomiting, nausea, and chronic damage to the immune system, liver, and kidney (Barac, 2019a). Patulin is very stable in low values of pH near four, and ripened fruits are ideal for their production and biosynthesis. It is mainly found in apple fruit and processed products (Kumar et al., 2018).

### 3.1 Patulin producing fungi

Fungi produce the patulin in different fruits, including *Aspergillus clavatus*, *A*. *giganteus*, *A*. *terreus*, *P. coprobium*, *B*. *nivea*, *Paecilomyces variotii*, *P. clavigerum*, *P. concentri-cum*, *Byssochlamys fulva*, *P. dipodomyicola*, *P. expansum*, *P. roqueforti*, *P. sclerotigenum*, *P. vulpinum*, *Penicillium carneum* and *P. glandicola* (Bodinaku et al., 2019; Paramastuti et al., 2021). However, *P. expansum*, The source of blue mould in apples and apple-related products, is the most prevalent and significant patulin producer (Piqué et al., 2013; Zaied et al., 2013). Although the optimum temperature for *P. expansum* growth is 25°C, it can also survive at -3°C. Patulin production decreases as the temperature decreases up to freezing temperature (0-4°C), and moisture content must be in the range of 0.82-0.83 for spore formation (Sommer et al., 1974; Hasan, 2000). In addition, *P. expansum* can grow in low O_2_ atmospheric concentrations and be found at as low as 2%. Furthermore, fruits’ physical and chemical properties, such as strength, flesh firmness, skin thickness, sugar content, pH, and antimicrobial compound presence, also affect patulin formation.

### 3.2 Patulin contamination on fruits and derived products

Although patulin impacts a variety of foods, the most pervasive toxic effect was seen in apples, as investigated by numerous researchers. A problem for fruit and goods derived from the fruit is the poisonous metabolite patulin produced by *Penicillium expansum*, which can contaminate many different foods. As a result, solutions that are affordable and successful are required to get rid of patulin and ensure food safety. Consuming mouldy and seemingly clean but fungus-infected products increases the danger of consumers being exposed to patulin. This could be explained by the fruit’s physicochemical properties, which encourage *P. expansum* growth and include factors like water activity and pH (Pleadin et al., 2019). Additionally, the genetic makeup of the crop, which affects its capacity for wound healing and susceptibility to infection, also impacts patulin production. In research by (Janisiewicz et al., 2008; Pleadin et al., 2019), Several global studies have been conducted to determine the levels of patulin contamination in apple juice and apple juice concentrates. While removing rotting or damaged fruit can decrease the amount of patulin in juices, it cannot be completely eliminated due to the diffusion of the mycotoxin into the fruit’s nutritional components. Studies have shown that the highest concentrations of patulin are typically found within 1cm of the injured area (Marín et al., 2011). *P. expansum*, also known as blue mould, is the primary culprit responsible for the presence of patulin in decayed apples as well as apple-based products like juices, jams, and ciders. Additionally, patulin contamination can also occur in other fruits such as plums, peaches, strawberries, apricots, and kiwifruits. (Neri et al., 2010; Sadok et al., 2018). To regulate patulin contamination in apple products like juice, cider, and puree, the European Union has established a maximum limit of 50 µg/kg. A study conducted in 2016-2018 found that patulin levels in apple juice and apple-based baby food were below the EU limit in most samples, but some exceeded the limit in some countries, such as Poland and Italy (Gaugain et al., 2020). The FDA has established a guidance level of 50 µg/kg for patulin in apple juice and apple juice concentrate, similar to the maximum limit set by the European Union. A survey conducted in 2015-2016 found that the majority of the tested apple juice samples (95%) were below the FDA guidance level, but some imported apple juice samples had higher levels (Biango-Daniels et al., 2019). Patulin contamination in fruits and their derived products has been thoroughly reported in China. A study conducted in 2015-2016 found that patulin levels in apple juice and apple puree samples from different regions of China ranged from below the detection limit to 189.0 µg/kg (Córdova et al., 2019). Similarly, previous studies also found that patulin levels in apple juice samples from Turkey ranged from below the detection limit to 258.2 µg/kg, with some samples exceeding the EU limit (Gottardi et al., 2016) (**Table 1**).

**Table 1:** Worldwide natural occurrence of *Alternaria* and patulin mycotoxins in fruits and derived products.

Fruit & Products	Mycotoxin	Country	Positive samples/total%	Range μg/kg μg/L	References
Apple	*Alternaria *	China	29(27.88)	0.08–6	Li et al., 2020b
Apple juice	*Alternaria *		41	2.20–3.10	–
Apple James	*Alternaria *		17	4.42-10.10	–
Apple vinegar	*Alternaria *	China	34	14.5	Li et al., 2020b
Apricot juice	*Alternaria *	Germany	14/20	1.95–3.97	–
Grape juice	*Alternaria*		4	50	Zwickel et al., 2016
Citrus juice	*Alternaria*		1/1	2.04	–
Grape juice	*Alternaria*		7/8	1.58-6.44	–
Apples	*Alternaria *	Netherland	1/11	29	Sanzani et al., 2016
Fruits dried	*Alternaria *	Switzerland	9	<2–17.2	Mujahid et al., 2020
Tomato,	*Alternaria *	Belgium	23/27	<3.5-31	Gotthardt et al., 2019
Rotten mandarins	*Alternaria *	Italy	–	1000–5200	Logrieco et al., 2002
Fruit juices,	patulin	Iran	–	50	
Apple, juice	patulin	south Korea	3/24	2.8–8.9	Wie et al., 2010
Apple	patulin	Czech	5/6	3.8-28.4	–
Strawberry	patulin		0/3	>0.5	–
Pear	patulin		2/3	11.3-28.9	Veprikova et al., 2015
Apple juice	patulin	Qatar	20/20	5.8-82.2	Hammami et al., 2017
Peach	patulin	Italy	2/30	> 10	–
Pear	patulin		25/39	>10	Spadaro et al., 2008
Mixed juice	patulin	Tunisia	17/34	10/55.7	Vaclavikova et al., 2015
Apple with soya	patulin	Spain	37	49.9	Sargenti and Almeida, 2010
Pear marmalade	patulin	Argentina	1/6	44676	Sadok et al., 2019b
Salad of mixed fruits	patulin	Belgium	1/100	–	Van de Perre et al., 2014
Apricot	patulin	Thailand	4/10	6.3	–
Peach	patulin		3/8	5.6	–
Grape	patulin		7/18	3.5	Puangkham et al., 2017
Pineapple	patulin	Malaysia	1/17	33	–
Lychee juice	patulin		1/17	13	Lee et al., 2014

Several species of *Aspergillus* and *Penicillium* produce patulin. In apples, the apple-rotting fungus *P. expansum* is the primary producer of patulin (Şenyuva and Gilbert, 2008). Patulin was found mainly in apples and sometimes in fruits like pears, apricots, and peaches, and mainly in rotten fruit parts Turkish visibly moulded and dried figs (Drusch and Ragab, 2003; Cheraghali et al., 2005; Karaca and Nas, 2006; Şenyuva and Gilbert, 2008). Numerous surveys have been carried out globally to investigate the levels of patulin contamination present in apple and apple juice concentrates (Cheraghali et al., 2005; Murillo-Arbizu et al., 2009). While removing rotten or damaged fruit may reduce the amount of patulin in juices, it is not possible to completely eliminate this mycotoxin as it can spread to healthy parts of the fruit (Gudmundsson et al., 2009). Eating food products that are infected with fungus, even if they appear visually clean, can increase the risk of exposure to patulin for consumers. This is because mycotoxin can accumulate not only in the visible lesion but also in other parts of the spoiled fruit (García-Cela et al., 2012; Sanzani et al., 2016). It is important to monitor patulin contamination levels in fruits and related products, including juices, purees, ciders, jams, marmalades, vinegar, and dried fruits, and to establish accepted levels for these mycotoxins (Zhu, 2014; WANG et al., 2018). According to recorded literature, the concentration of patulin in apples that are infected with the toxin can range from 8.8 to 120.4 µg kg^-1.^ Ritieni tested six samples of apple puree and found that three of them had patulin concentrations ranging from 15.9 to 74.2 µg kg^-1^. However, only one of the samples met the European Union’s standards for patulin levels, which stipulate that the mycotoxin should not exceed 25 µg kg^-1^ (Camaj et al., 2018). Four out of eight apple puree samples contained 22-221µg kg^-1^ of patulin, according to Funes and Resnik (Zhang et al., 2020b). In addition, the fungi that produce patulin contaminate other fruit products, Pears are also a source of this mycotoxin and have been found to have high levels of patulin, exceeding the recommended limit set by the European Union (Karlovsky et al., 2016a). Because the loss of patulin during industrial processing is relatively tiny, apple juices are mainly associated with patulin contamination (Zhang et al., 2020b). In China, patulin was detected in 19 out of 30 baby food products analyzed, with the maximum concentration of the mycotoxin reaching 67.3 µg/kg (Yuan et al., 2010). Other products from apples tested in Italy contained a significant amount of patulin. This emphasizes the importance of developing preventative actions and food surveillance programs to better protect children from toxin exposure. Patulin-producing fungi can contaminate various fruits other than apples, serving as another source of this mycotoxin. It is becoming more and more crucial to regularly check the levels of patulin present in colored fruits, such as hawthorns, red grapes, plums, sour cherries, as well as various types of berries like strawberries, raspberries, blueberries, and blackberries (Vaclavikova et al., 2015; Vickers, 2017; Iqbal et al., 2018; Wu et al., 2019).

### 3.3 Biosynthesis of patulin

**Figure 1:**
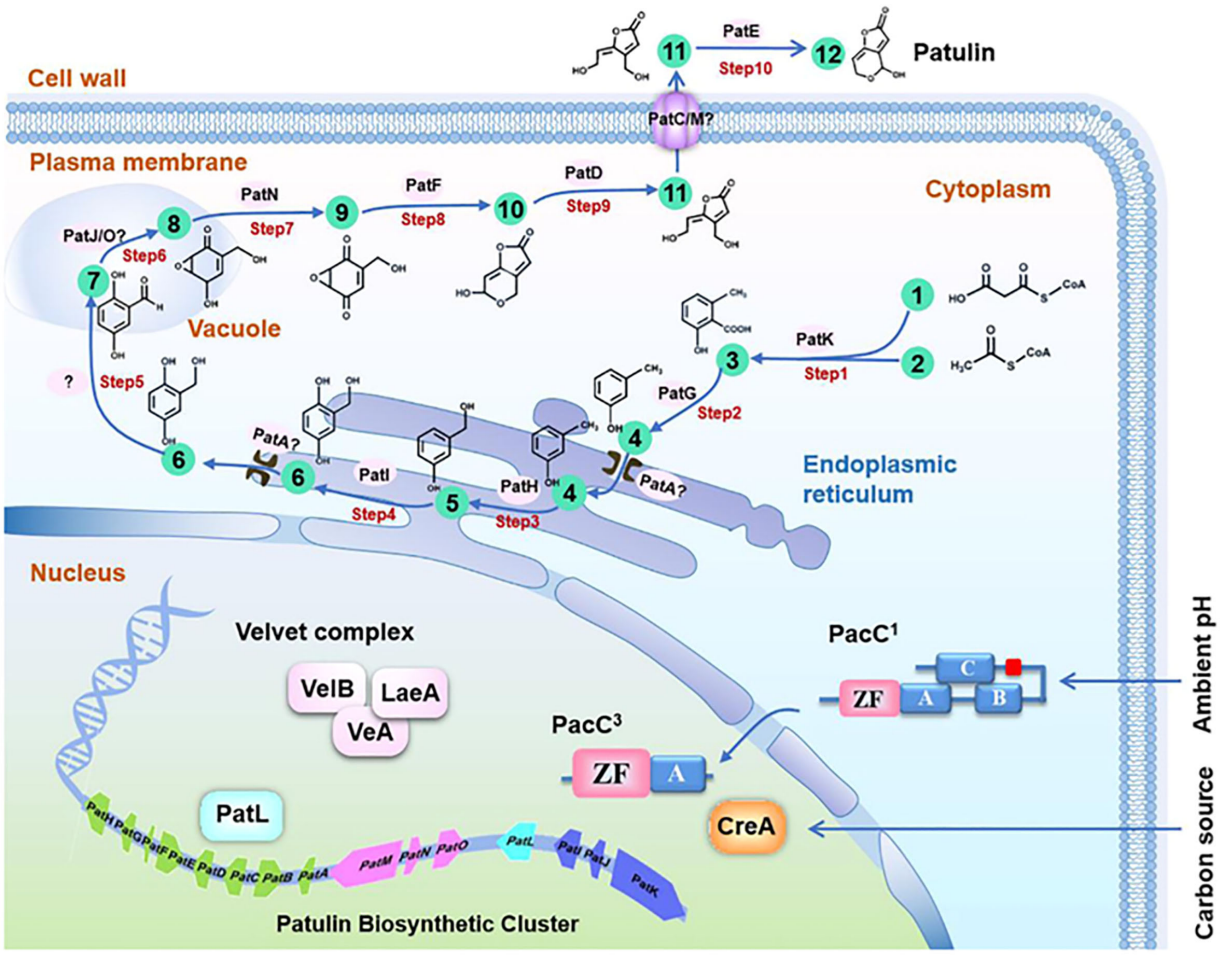
Molecular basis and regulation of the patulin biosynthetic pathway in *Penicillium expansum*. The patulin biosynthetic pathway consists of a 10-step reaction and 12 metabolites. 1, Acetyl CoA; 2, Malonyl CoA; 3: 6-Methylsalicylic acid; 4: m-Cresol; 5: m-Hydroxybenzyl alcohol; 6: Gentisyl alcohol; 7: Gentisaldehyde; 8: Isoepoxydon; 9: Phyllostine; 10: Neopatulin; 11: E-ascladiol; 12: Patulin. (Li et al., 2020a).

The biosynthesis of patulin involves several enzymatic reactions that occur in the fungal cell. The first step is the condensation of two molecules of acetyl-CoA to form 6-methylsalicylic acid (6-MSA), which is catalyzed by the polyketide synthase (PKS) enzyme. Then, 6-MSA is converted into patulin by a series of reactions that involve oxidation, decarboxylation, and esterification reactions. Patulin is a secondary metabolite derived from polyacetate, and its metabolic pathway has been extensively studied utilizing cell-free extract and kinetic pulse-radiolabelling systems (Tian et al., 2018). The first limitation on patulin production has been identified as the inactivation of 6-MSA synthetase (Hitschler and Boles, 2019). Loss of 6-MSA synthetase is a selective process because *P. urticae* has a highly similar fatty acid synthetase (Moake et al., 2005). A study was conducted to investigate the stabilization of 6-MSA synthetase through the treatment of reaction mixtures containing nicotinamide adenine dinucleotide phosphate (NADPH) cofactor, Acetyl-CoA, and malonyl CoA with the reducing agent dithiothreitol and the proteinase inhibitor phenylmethylsulfonyl fluoride. The results demonstrated that the treatment effectively stabilized 6-MSA synthetase. This suggests that proteolysis and conformational integrity play a crucial role in 6-MSA synthetase regulation (Joshi et al., 2013). The activity of 6-MSA decarboxylase converts 6-MSA into m-cresol in the next stage of patulin biosynthesis (Kong et al., 2018). Further, m-cresol 2-hydroxylase converts m-cresol to m-hydroxy benzyl alcohol (Mahato et al., 2021). There is ongoing debate regarding the next step in the biosynthetic pathway of patulin, with two proposed mechanisms. However, both mechanisms agree that m-hydroxy benzyl alcohol eventually transforms into gentisaldehyde. Therefore, the conversion of m-hydroxy benzyl alcohol to gentisaldehyde is considered a crucial step in the patulin biosynthetic pathway (Yli-Manila and Gagkaeva, 2018). However, the intermediary between these two compounds is thought to be either gentisyl alcohol or benzyl alcohol (Lubbers et al., 2019). The exact mechanism of patulin biosynthesis in *P. expansum* is still under investigation. However, recent studies have identified several genes involved in patulin biosynthesis, including the *PKS* gene (*pksJ*), the oxidoreductase gene (*patG*), and the methyltransferase gene (*patK*). These genes are clustered together in the fungal genome and are regulated by a pathway-specific transcription factor (*patL*). Additionally, environmental factors such as temperature, pH, and nutrient availability can affect patulin production in fungal cells. For instance, studies have shown that low pH conditions and low temperatures can increase patulin production in *P. expansum*, while high temperatures and nutrient-rich conditions can decrease patulin production (Liu and Wu, 2010; Li et al., 2019) (**Figure 1**).

### 3.4 Health impact of patulin mycotoxin

The assessment of the health risks posed by patulin to human beings is based on many studies conducted over the past 50 years. Patulin causes various acute, chronic, and cellular levels in humans (Fung and Clark, 2004). Ingestion of patulin can lead to a range of adverse effects, including agitation, convulsions, dyspnea, pulmonary congestion, edema, ulceration, hyperemia, distension of the gastrointestinal tract, intestinal hemorrhage, degeneration of epithelial cells, inflammation of the intestines, vomiting, and damage to the gastrointestinal and kidney tissues (Celli et al., 2009; Pal et al., 2017; Aslam et al., 2021). Additionally, chronic health risks associated with patulin consumption include neurotoxic, immunotoxin, immunosuppressive, genotoxic, teratogenic, and carcinogenic effects (Abrunhosa et al., 2016; Boussabbeh et al., 2016; Ülger et al., 2020; Cimbalo et al., 2020; Ráduly et al., 2020).

Patulin has been associated with a range of health impacts, including neurotoxicity, immunotoxicity, and carcinogenicity. Patulin mycotoxin is recognized as a potential health risk worldwide, and regulatory agencies have established maximum limits for patulin in fruit products to ensure that consumers are protected. Consumers should follow safe food handling practices, including checking for spoilage and discarding any fruits or juices that show signs of spoilage or mold growth. The incidence of patulin contamination is a global problem, mainly for the primary producers of apples and apple-based products, such as the USA, EU, and China (USDA, 2018). The Food and Drug Administration (FDA) of United States has set a maximum limit for patulin in apple juice and apple juice concentrate at 50 parts per billion (ppb). Exposure to high levels of patulin has been linked to liver damage and immunotoxicity in animal studies. However, the risk of adverse health effects from patulin in apple products is considered low for the general population, as most people consume these products in moderation (FDA et al., 2005). The European Commission has set a maximum limit for patulin in apple juice and apple juice concentrate at 50 ppb, the same as in the US. In addition, the EFSA has conducted risk assessments of patulin in other food products, such as pears, grapes, and dried fruits, and has concluded that exposure to patulin from these sources is also unlikely to pose a health risk for the general population (Capriotti et al., 2010). China has also set a maximum limit for patulin in apple products at 50 ppb. In recent years, there have been several reports of high levels of patulin in apple products in China, leading to concerns about food safety. The Chinese government has taken measures to improve the quality and safety of apple products, including strengthening regulations and increasing inspections of apple processing facilities (Capriotti et al., 2010). Health Canada has set a maximum limit for patulin in apple juice and apple juice concentrate at 50 ppb, consistent with the US and European limits. Health Canada has also conducted risk assessments of patulin in other food products, such as pears and cherries, and has concluded that exposure to patulin from these sources is unlikely to pose a health risk for the general population (Mendonca et al., 2020). The maximum allowable limit for patulin in apple juice and apple juice concentrate is set at 50 parts per billion (ppb) by the Australia New Zealand Food Standards Code, which aligns with the limits established in other nations. To ensure adherence to these regulations, the Australian government monitors the levels of patulin in apple products (Ghosh, 2014). Studies in India have shown that patulin can cause neurotoxicity, including oxidative stress and changes in neurotransmitter levels, and may also have genotoxic and carcinogenic effects. However, there is currently no specific regulatory limit for patulin in fruit products in India (Diao et al., 2019). A study conducted in Brazil found that patulin levels in apple juice samples were generally low, but higher levels were found in some samples. The study also found that patulin was not significantly associated with the occurrence of gastrointestinal symptoms in children (Dias et al., 2019). Similarly, a study conducted in Turkey found that patulin levels in apple juice and apple-based products were generally low, but higher levels were found in some samples (**Table 2**). The study also found that patulin was not significantly associated with the occurrence of gastrointestinal symptoms in adults (İçli, 2019).

**Table 2:** Methods for detecting PAT using biosensors.

Biosensor Methods	Sensitivity/LODs (μg/L)	Linearity (μg/L)	Pattern of Recognition	Mode/Type of Transmission	Reference
The use of ZnO nanorods on aptamer-based voltammetric patulin assay.	2.7×10^-4^	5.0×10^-4^-50	Self-assembled thio-modified aptamer complex on AuNPs	ZnO nanorods (ZnO-NRs) and chitosan composite modified gold electrode	Liu et al., 2018
Carbon dots, chitosan and gold nanoparticles have been added to a novel molecularly imprinted electrochemical sensor.	1.2 × 10^–4^	5 × 10^–4^–1.5 × 10^-1^	Modified MIP cavity absorption	Electrochemical detection	Guo et al., 2017
Enzyme biosensor with conductometry	<7.7 × 10^-2^	<1.5 × 10^-2^-7.7 × 10^-3^	Enzymes (Urease Inhibition)	Interdigitated gold electrodes in a differential pair	Soldatkin et al., 2017
Method of near-infrared fluorescence assay	< 6.0 × 10-2	0.0-3.7 × 10^-1^	Anti-PAT antibodies conjugated to fluorophores	Fluorescence in the near infrared (NIR)	Pennacchio et al., 2015
Biochip based on surface plasmon resonance	1.5 × 10^-2^	0.0-12.3	Antibodies compete with anti-patulin	Resonance of surface plasmons (SPR)	Vickers, 2017
A colorimetric DNA apt sensor.	4.8 × 10^-2^	5.0 × 10^-2^–2.5	DNA aptamer	Colorimetric detection	Jin et al., 2019
Immune system-based sensor (biosensor) on nanoporous silicon sensor (biosensor)	–	–	Immune system based on nanoporous silicon	Single-crystal silicon square wafers doped with boron	Starodub and Slishek, 2013
Aptasensor Impedimetric	2.8 × 10^–3^	25 × 10^-3^–1	A carbon-based electrode -immobilized aptamer interaction	Electrochemical detection	Khan et al., 2019
Molecularly imprinted electrochemical sensing platform (thionine)	1.0 × 10^–3^	2.0 × 10^–3^–2	Thianine tailing surface	Electrochemical detection	Khan et al., 2019
Quartz microbalance sensor based on molecular sol-gel polymer (MIP)	3.1	7.5-60	Sol-gel molecular polymer (MIP) Changing QCM frequency	–	Chen et al., 2016
Surface-impressed gold Surface-enhanced nanoparticles Scatter sensor (MIP-SERS) Sensor) Sensor	8.3×10^-4^	1.1 × 10^–3^–7.7	Selectivity of molecular polymer imprinted	Electrochemical detection	Wu et al., 2019
Molecularly imprinted polymer surface Capped ZnS quantum dots Mn-doped as a nanosensor phosphorescent	49.3	1.0 × 10^–3^–66.3	6-hydroxynicotinic acid affinity (6-HNA)	Detection of phosphorescence quenching	Zhang et al., 2017b

Patulin has been shown to have cellular effects such as plasma membrane disruption (Li et al., 2019), protein synthesis inhibition of Na+-coupled amino acid (Schilling and Eder, 2004), disruption of transcription and translation (Rutkowski et al., 2015; Zhu et al., 2019), inhibition of DNA synthesis (Varzatskii et al., 2018) as well as the inhibition of T-helper type 1 cells that produce interferon (Chai et al., 2010). Patulin damages cells by forming adducts with thiol-containing cellular components like glutathione and cysteine-containing proteins (Pal et al., 2017). Patulin is toxic to many enzymes with a sulfhydryl group in their active site. A recent study revealed that patulin inhibits ATPase, which is Na+ K+ dependent RNA polymerases (Lee et al., 2019) and the synthetase of aminoacyl tRNA (Cervettini et al., 2020). Furthermore, exposure to patulin results in the loss of free glutathione in living cells (Pal et al., 2017). Recent research revealed that the exogenous cysteine and glutathione treatment prevents patulin toxicity in the intestinal epithelium (Saleh and Goktepe, 2019). Patulin has also been shown to promote intramolecular and intermolecular protein cross-linking. This reaction favours cysteine’s thiol group, but it also happens with lysine and histidine’s side chains and -amino groups (Qiu et al., 2020). Other studies have also observed patulin’s reactivity with NH_2_ groups (Plunkett et al., 2019). Patulin has also been shown to inhibit protein prenylation, a necessary posttranslational protein modification involved in activating many proteins, including many oncogenes, such as Ras, that must be prenylated to function appropriately (Zheng et al., 2017).

## 4 *Alternaria* mycotoxins

The *Alternaria* genus was first described in 1816 (Woudenberg et al., 2013). *Alternaria* species belong to the phylum Ascomycota, commonly called sac fungi. It is saprophytic and parasitic and can decompose organic matter largely. It may also become an opportunistic pathogen sometimes causing various diseases in cereal crops, ornamentals, vegetables, and fruits (Thomma, 2003). *Alternaria* spp. produce toxins as secondary metabolites that develop cancer and are mutagenic, causing health disorders in animals and humans (Pastor and Guarro, 2008). Moreover, its spores are airborne allergens that are problematic in certain situations (Kilic et al., 2020). Besides, *Alternaria* pathogenic species cause blight, leaf rot, and leaf spot diseases in plants associated with both host-specific and non-host-specific toxins (Yamamoto et al., 2014; Meena and Samal, 2019), causing black spots in various vegetables and fruits during the post-harvest period of storage and marketing (Thomma, 2003). *Alternaria* mycotoxins are considered emerging mycotoxins because they have been increasingly recognized as a potential health hazard in recent years. *Alternaria* fungi are known to produce a wide range of mycotoxins that can contaminate a variety of fruits and their products. Exposure to these mycotoxins has been associated with various adverse health effects, including cancer, allergies, and other toxicities. The classes of *Alternaria* mycotoxins include, alternariol (AOH), alternariol monomethyl ether (AME), tentoxin, altenuene, alternapyrone, and aurasperone A. Several studies conducted on the occurrence of *Alternaria* mycotoxins in fruits in different countries. Studies conducted in Italy found that *Alternaria* mycotoxins were present in a variety of fruits, including apples, pears, and grapes. The most common mycotoxins detected were AOH and AME (Zhou and Hu, 2018; Ye et al., 2019). A study in China found that *Alternaria* mycotoxins were present in a variety of fruits, including apples, pears, and peaches. The most common mycotoxin detected was AOH (Ji et al., 2022). A study in Iran found that *Alternaria* mycotoxins were present in pomegranates, with the most common mycotoxin detected being tentoxin (Vidal et al., 2019). A study in Spain found that *Alternaria* mycotoxins were present in strawberries, with the most common mycotoxin detected being AME. A study in Brazil found that *Alternaria* mycotoxins were present in mangoes, with the most common mycotoxin detected being AOH (Qiao et al., 2018; Cinar and Onbaşı, 2019). It is also important to note that the occurrence of *Alternaria* mycotoxins in fruits can vary depending on a number of factors, including the type of fruit, the location where it was grown, and the environmental conditions during cultivation and storage (**Table 1**).

### 4.1 *Alternaria* mycotoxins-producing fungi

The naturally occurring secondary metabolites known as “*Alternaria* mycotoxins” are produced by toxigenic micro-fungi that grow on crops (Nesic et al., 2014; Tralamazza et al., 2018). Different species of *Alternaria* can produce mycotoxins, including *A. scripinfestans*, *A. botrytis*, *A. oudemansii*, *A. leptenallea*, and *A. alternata* (Buczacki, 2003). The most common and important species is *A. alternata*, which produces mycotoxins and grows on cereal crops, vegetables, fruits, olives and sunflower seeds (Scott, 2001; Scott, 2004). Some other species were also called etiologic agents, including *A. tenuissima*, *A. longipes*, *A. infectoria*, *A. dianthicola*, and *A. chlamydospora* (Bartolome et al., 1999; Ferrer et al., 2003; Robertshaw and Higgins, 2005; Nulens et al., 2006). The morphology of these species is different yet challenging to identify and is a pretty daunting task to do (Sutton et al., 2009). To minimize the risk of exposure to *Alternaria* mycotoxins, it is important to store food properly, discard any moldy or damaged food, and avoid consuming foods that are known to be contaminated with these toxins. Additionally, agricultural practices that promote healthy plant growth and reduce fungal contamination can help to prevent the growth of *Alternaria* fungi and the production of mycotoxins in crops.

### 4.2 *Alternaria* mycotoxins contamination on fruits and derived products

*Alternaria* can produce mycotoxins, which are toxic compounds that can contaminate food and feed products (Müller et al., 2018). The mycotoxins produced by *Alternaria* toxin can cause adverse health effects in humans and animals if ingested in sufficient quantities. One of the most commonly produced *Alternaria* mycotoxins is AOH, which has been detected in various fruits and their derived products such as apple juice, grape juice, tomato sauce, and dried fruits (Solfrizzo, 2017). AOH has been shown to be genotoxic, carcinogenic, and immunosuppressive in animal studies, and its presence in food products has raised concerns about its potential health effects on humans. Another *Alternaria* mycotoxin that has been found in fruits and their derived products is alternariol monomethyl ether. AME has been detected in apple juice, grape juice, tomato products, and dried fruits. Like AOH, AME has been shown to be genotoxic and carcinogenic in animal studies. Other *Alternaria* mycotoxins that have been detected in fruits and their derived products include TeA, ALT, and ATX-I. TeA has been found in apple juice, grape juice, and tomato products, while ALT and ATX-I have been detected in apple products (Houbraken et al., 2010). Much research revealed that *Alternaria* pathogenicity is higher in fruits and leaves than in other plant parts (Harteveld et al., 2014). Secondary metabolites of *Alternaria* spp. may cause other plant diseases such as black tomato mould, the grey/black mould of citrus, olive black rot, apples, and carrot black rot (Logrieco et al., 2009). The presence of *Alternaria* mycotoxins in fruits and their derived products is a concern for food safety and human health. To mitigate the risk of mycotoxin contamination, it is important to implement good agricultural and manufacturing practices, including proper storage and handling of fruits and their derived products, as well as monitoring and testing for mycotoxin contamination (Li et al., 2020b).

*Alternaria* fungi are parasitic on plants and may cause fruit and vegetable spoilage during transportation and storage. *A. Alternata* is capable of producing a range of mycotoxins, such as alternariol dibenzo-α-pyrones, tenuazonic acid (a tetramic acid), ATX-I and II, monomethyl ether, and altenuene. It is important to note the variety of mycotoxins produced by *A. Alternata* (Yang et al., 2014). *Alternaria* toxins AME and AOH can be produced within a range of 5-30°C and a water activity (aw) range of 0.98-0.90. However, at the lower end of this a_w_ range (i.e., 0.90), very few mycotoxins are produced. The minimum aw required for *A. alternata* conidia to germinate is 0.85. In contrast, wheat growth requires an a_w_ of at least 0.88 in extract agar at 25°C. As a result, the limiting aw for detectable mycotoxin production is slightly higher than for growth, with optimum production above 0.95 a_w_ (Magan et al., 1984). *Alternaria* mycotoxins can be found in various fruits, vegetables, and grains (Scott, 2001). AOH and AME are among *Alternaria* main mycotoxins, naturally reported as occurring in different fruits that are infected, including mandarins, oranges, melons, apples, lemons, and various berries (Scott, 2001; Drusch and Ragab, 2003; Scott et al., 2006). Tenuazonic acid was also present in these citric at high levels (Magnani et al., 2007). Monomethyl ether alternariol and alternariol were detected in tangerines in Brazil with and without symptoms of spot disease *Alternaria*; levels of these flavedo mycotoxins varied between 0.90 and 17.40 µg/kg **Table 1**. In albedo (mesocarp) tissues, neither AOH nor AME has been detected, suggesting that the flavedo is working as a barrier to these substances. The natural occurrence of *Alternaria* toxins in processed foods interests from a human health point of view. AOH and AME were detected in most of the fruit’s juices with shallow (<1.5 µg/L) levels, except for apple, grape juice and red wine (Drusch and Ragab, 2003; Scott et al., 2006). The natural occurrence of AOH and AME in apple juice was reported at levels ranging from 0.04 to 2.40 µg/L and 0.03 to 0.43 µg/L **Table 1** **t1**, respectively; other fruit juices, such as grape juice, had levels of 1.6 and 0.23 µg/L for AOH and AME, respectively, 5.5 and 1.4 µg/L for prune nectar, and 5.6 and 0.7 µg/L for cranberry nectar low levels of raspberry juice have also been detected (Lau et al., 2003). Both mycotoxins have been found in apple juice concentrates from Spain 50% of the samples were analyzed as natural contaminants. Levels of the AOH were in the range of 1.35–5.42 µg/L. AME was present in most cases only at trace levels, and the highest number detected in one sample was 1.71 µg/L (**Table 1**). AOH occurs very often at low levels in red wine (Scott et al., 2006) AOH was found in 13/17 Canadian red wines at a level of 0.03–5.02 µg/L and in 7/7 imported red wines at a level of 0.27–19.4 µg/L, **Table 1** accompanied by lower AME concentrations. White wines contained a small amount of AOH/AME (≤1.5 ng/mL). As far as we know, there are no studies on the co-occurrence of *Alternaria* toxins in fruit with other mycotoxins.

### 4.3 Biosynthesis of *Alternaria* mycotoxin

**Figure 2:**
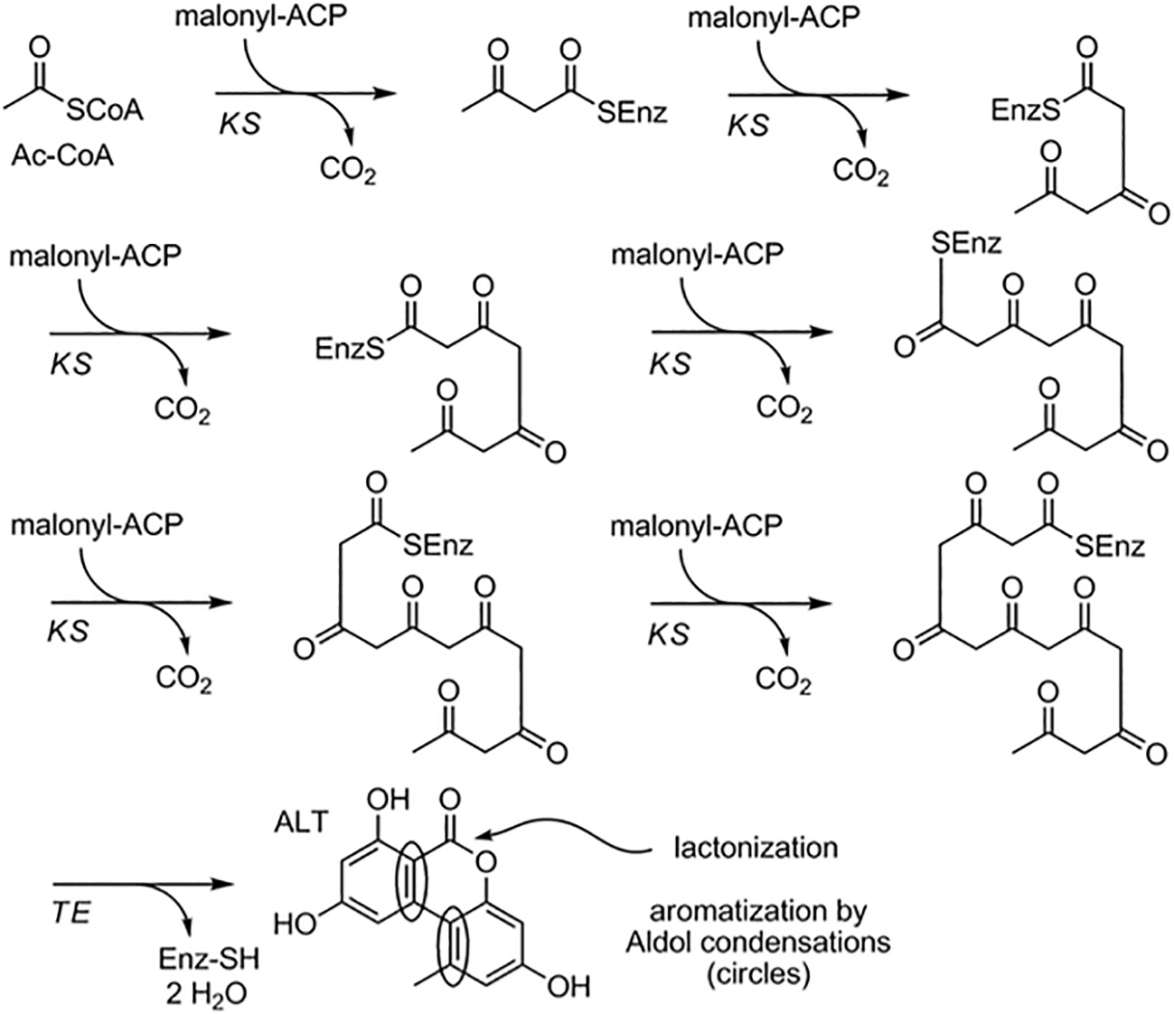
Proposed biosynthetic pathway for alternariol and alternariol-9-methyl ether.(Saha et al., 2012).

The biosynthesis of *Alternaria* toxin metabolites is a complex process involving many enzymes and pathways. One well-studied example of *Alternaria* toxin metabolite biosynthesis is the production of the mycotoxin AOH. AOH is a hydroxylated form of the precursor AME and is synthesized through a series of enzymatic reactions. The biosynthesis of AOH in *Alternaria* toxin involves a *PKS* gene cluster, which includes genes for a *PKS*, a ketoreductase (KR), an enoyl reductase (ER), and a cytochrome P450 monooxygenase (CYP). The *PKS* gene cluster produces a polyketide intermediate that is subsequently modified by the KR and ER enzymes to form the AOH precursor, AME. Finally, the CYP enzyme catalyzes the hydroxylation of AME to produce AOH. The biosynthesis of AOH has been extensively studied in *A. alternata*, and the *PKS* gene cluster responsible for AOH biosynthesis has been identified and characterized (Darma et al., 2019). Consequently, *Alternaria* toxin metabolite biosynthesis is the production of tenuazonic acid (TeA), a mycotoxin produced by many species of *Alternaria*. TeA is synthesized from a precursor molecule, 1,8-dihydroxynaphthalene (DHN), through a series of enzymatic reactions. The biosynthesis of TeA in *Alternaria* toxin involves a nonribosomal peptide synthetase (NRPS) gene cluster, which includes genes for an *NRPS*, a *PKS*, and a thioesterase (TE)(Yun et al., 2015). The NRPS enzyme catalyzes the formation of a peptide bond between two amino acids, while the PKS enzyme produces a polyketide intermediate. The TE enzyme cleaves the peptide-polyketide intermediate, leading to the formation of DHN, which is then modified to form TeA. The biosynthesis of TeA has been studied in several species of *Alternaria*, including A. *alternata*, and the *NRPS* gene cluster responsible for TeA biosynthesis has been identified and characterized (Yun et al., 2015) (**Figure 2**).

### 4.4 Health impact of *Alternaria* mycotoxin

The toxicity of *Alternaria* toxins in animals and humans has received insufficient attention compared to other commonly reported mycotoxins. While AOH and AME are not particularly acutely toxic to animals but can still cause organ hemorrhages, some animal species, like dogs, are highly toxic to TeA. For instance, they generate internal haemorrhage in the chicken while they reduce feed efficiency in mice (Agriopoulou et al., 2020). Three mammalian regular cell lines, 3T3 mouse fibroblasts, Chinese hamster lung cells, and human hepatocytes are the most sensitive to TeA cytotoxic effects on cultured cells, which include lowering total protein concentrations and suppressing proliferation (Song et al., 2020). AME and AOH are genotoxic, though previous research has shown that AOH is more genotoxic than AME in cultured human colon carcinoma cells while causing fetal oesophagal squamous (Aichinger et al., 2017). Even though individual mycotoxin concentrations are usually within permissible limits, high co-contamination rates could harm human and animal health. It is crucial to thoroughly evaluate the toxicological traits of individual mycotoxins and co-occurring mycotoxins. *Alternaria* mycotoxins produced can be harmful to human health and to ensure the safety of fruits and their derived products, many countries have established regulations on the maximum permitted limits of *Alternaria* content in these products (**Table 2**).

## 5 Detection technology of patulin and *Alternaria* mycotoxins in fruits and derived products

### 5.1 Biosensors detection

Patulin in different food commodities has historically been determined using several chromatographic techniques, such as gas chromatography, high-performance liquid chromatography, thin-layer chromatography, and others, have been used to analyze patulin levels in different food commodities (Kaur and Singh, 2020). These are sensitive and targeted methods. However, they require costly equipment and highly skilled operators (Kaur and Singh, 2020). Biosensor technologies, as opposed to chemical methods, have recently been developed to provide some “cleaner” patulin detection techniques for apple juice. Selectivity, which allows for immediate detection of the analyte with little or no pretreatment, is an undeniable advantage of biosensors over traditional food analysis methods. They do not necessitate highly trained personnel and are simple to operate (Ngolong Ngea et al., 2020). Biosensor technologies use a specific bio-recognition component and a transducer to process the signal. The affinity of the bio-recognition component to the patulin molecule will determine its efficacy. The sensitivity of biosensors will depend on their capacity to detect even the weakest modification signal, usually an electrochemical signal, following patulin bio-recognition. We have included some recent examples of biosensors below. The competitive immunoassay is an intriguing method for investigating patulin in food. An innovative strategy has been developed by combining immunological recognition of patulin with a surface plasmon resonance optical procedure to create polyclonal antibodies (SPR) (Tittlemier et al., 2021). A laser beam initiated interactions between the test and targeted molecular particles on the biochip’s gold surface. This induction causes a shift in resonance conditions and, as a result, a subtle but noticeable change in reflectivity. This method was described as a cost-effective and efficient immunoassay for determining patulin. According to Vickers’ study on apple juice, the LODs in this test was found to be 1.54 µg/L. It should be noted that patulin was not detected prior to the sampling process (Vickers, 2017). A new fluorescence polarization method using near-infrared (NIR) fluorescence sensors was also been developed that showed a great potential in fluorescence detection and measurement (Víctor-Ortega et al., 2013; Pennacchio et al., 2015). The increase of emissions of fluorescence polarization of a fluorescence-labelled patulin derivative, which binds to particular antibodies, is characterized. The LODs for the patulin ranges from 6 to 102 µg/L due to competition between the patulin and the fluorescence-labelled patulin derivative (Melinte et al., 2022). The proposed technique was based on the unique properties of crystal or quartz materials. Oriented antibodies that were tethered were immobilised using photonics on the gold-plated surface of a quartz-equipped microbalance. This biosensor identified patulin at a concentration of 21.56 µg/L. An extra antibody was added in a “sandwich procedure” to enable detection of nano-sized analytes measured by a microbalance. Furthermore, the goal is to develop a simple luminescent sensor capable of detecting patulin. Further, Zhang et al. (2017b) have created a nano-sensor based on manganese-doped ZnS quantum dots that use phosphorescence to discriminate patulin selectively. With a LODs of 49.31 µg/L, this nano-sensor can detect patulin from a concentration range of 66.22 to 1.001 µg/L. It can also differentiate patulin from other mycotoxins. Much of the recent research has focused on the problem of restoring biosensor activity after it has been used. As a result, Soldatkin et al. (2017) tracked patulin inhibitory action, and researchers created a conduct metric urease-based biosensor. This biosensor is suited for assessing patulin concentrations beyond 50 µg/L in apple juices because it has a relatively high patulin sensitivity, strong selectivity, and great signal repeatability. The presence of heavy metals, on the other hand, causes some problems. Patulin and other heavy metals can create strong covalent interactions with enzyme sulfhydryl groups. An oligonucleotide aptamer is a monoantennary DNA (or RNA) sequence. Aptamers are typically selected using a well-known method. The acronym SELEX (systematic evolution of ligands) stands for “systematic evolution of ligands.” There are ssDNA aptamers present (by exponential enrichment). It generally has a high affinity for patulin and interesting properties like easy synthesis and labelling, no immunogenicity, low production costs, high stability, affinity, and outstanding specificity in target binding. This chosen aptamer was later used as a selective component in a patulin detection method based on a polymerase chain reaction. Enzymatic substrate system with chromogenic properties. The result was that the colourimetric aptasensor provided a linear response, which was very impressive. The detection range is 5 102 to 2.5 µg/L, and the detection range is 5 102 to 2.5 µg/L. The limit was 4.8 102 µg/L (Wu et al., 2015). Lanthanide-doped rare earth-doped up-conversion nanoparticles (UCNPs) have gotten much attention as a technique to increase biosensor signal transmission (Loo et al., 2019). NIR-to-visible up-conversion nanoparticles (UCNPs) offer several advantages over traditional down-conversion luminescent devices. These include low auto-fluorescence background for improved signal-to-noise ratio, high photostability, low toxicity, high Stokes offsets, tunable fluorescence wavelength, and deep tissue infiltration. Biosensors linked to a transducing system that uses biological tools like enzymes, aptamers, and antibodies for recognition are employed to detect patulin in food. Aptamers are gaining popularity due to their remarkable ability to recognize patulin at low concentrations and modify their absorption properties, enabling detection at extremely low levels. They can be useful for on-line patulin control in the food industry. The main challenges of biosensors are the limited stability of the bio-recognition component (which affects the biosensors’ long-term storage stability), poor selectivity, especially in enzyme inhibition-based biosensors, and the high cost of antibodies when compared to synthetic recognition elements (Kaur et al., 2015; Burcu Aydın et al., 2020) (**Table 3**).

**Table 3:** Comparison of Liquid liquid extraction conditions used for fruit commodity preparation for patulin analysis.

Application	Sample size	Extraction solvent volume	Organic phase cleanup	Additional steps	Recovery spiking level	Reference
Fruit juice	50g^a^or 50mL	50 mL of EtAC (15 min)2 × 20 mL of EtAC	2 mL of 1.5% Na2CO3 + 5 mL of EtAC (5 min)	Filtration, evaporation, and solvent reconstitution	85.5–93.7% (50.0–200.0 μg mL^-1^)	Iqbal et al., 2018
Apple juice	5mL	20 mL of EtAC (15 min)	–	Reconstitution of the solvent, Evaporation	77.0–113.0% (10.0–1000.0 μg kg^-1^)	–
Fruit juices,	–	–	–	–	–	Hammami et al., 2017
Apple juice,	5 mL or 5 g^a^	0 mL of EtAC (10 min);2 × 20 mL of EtAC	2 mL of 1.5% Na_2_CO+ 5 mL of EtAC (3 min)	Solvent, evaporation filtration, reconstitution	86.5%(100.0–300.0 μg mL-1)	Zouaoui et al., 2015
Concentrate,	–	–	–	–	–	–
Appple jam	–	–	–	–	–	–
Apple juice	10mL	3 × 20 mL of EtAC(3 × 1 min)	4 mL of 1.5% Na_2_CO_3_+ 10 mL of EtACb	Filtering, evaporation, reconstitution of a solvent	86.0% (50 ug L^-1^)	
Juices of fruit,	10mL	3 × 20 mL of EtAC(3 × 10 min)	2 mL of 2% Na2CO3	SPE clean-up, evaporation solvent reconstitution	96.1–115.7%(20.0–50.0 μg L^-1^	Zaied et al., 2013
Fruit juices	5mL	2 × 10 mL of EtAC(2 × 1 min)	2 mL of 1.5% Na2CO3+ 5 mL of EtACb	Filtration	70.0–82.0%(50.0–150.0 μg L^-1^	Cho et al., 2010
Apple cloudy juice,	10g^a^	10 mL of EtAc: n-hexane(60: 40, v/v) (5 min)	15.0 g Na2SO4, 2 g NaHCO_3_	Clean-up of SPE, evaporation, solvent reconstitution, and filtration	53.0–74.0%(5.0–48.0 μg kg^-1^)	–
Apple puree,	–	–	–	–	–	Režek Jambrak et al., 2018
Apple clear, Concentrated juice	5g^a^	3 × 25 mL of EtAC(3 × 1 min)	10 mL of 1.5% Na2CO3(10 s) + 10 mL of EtAC (1 min) b	solvent reconstitution, evaporation	75.2–89.2%(10.0–500.0 μg kg^-1^)	Malachová et al., 2014

### 5.2 Molecular detection methods

Controlling patulin requires early detection of it-producing fungi (Mahunu et al., 2016). *Aspergillus clavatus*, *Aspergillus longivesica*, *Aspergillus* species, and *Aspergillus giganteus* are among the fungi that produce patulin. In the *Penicillium* genus, there are 13 species that produce patulin, including *P. vulpinum, P. sclerotigenum, P. paneum, P. marinum, P. griseofulvum, P. gladioli, P. glandicola, P. dipodomyicola, P. expansum, P. coprobium, P. concentricum, P. clavigerum, and P. carneum* (Mahunu, 2017). Polyphasic analysis of all *Byssochlamys* and related *Paecilomyces* species revealed that only *Byssochlamys Nivea* and a few strains of *Paecilomyces saturatus* could produce patulin (Fontaine et al., 2016). Given a large number of producers, biotechnology methods that identify the presence of patulin-producing fungi can assist in identifying the crucial areas that need to be controlled. PCR-based methods for detecting patulin -producing fungi strains in food samples can be used as a standard approach in agri-food HACCP procedures. The *IDH1* gene encodes the isoepoxydon dehydrogenase enzyme, which is required for patulin biosynthesis. In general, detection PCR protocols use high sensitivity and specificity to amplify the IDH1 sequence found in the genomes of some fungi species, such as *P. expansum* (Niessen, 2007; Delgado et al., 2021). However, some ingredients in complex foods inhibit PCR. These substances inhibit polymerase activity, which is necessary for DNA amplification, and cellular lysis, which is necessary for DNA extraction, capture, or degradation. Fortunately, a suitable nucleic acid extraction protocol could overcome this constraint (Aparicio et al., 2019). Regarding sensitivity, Delgado et al. (2021) developed RTi-PCR tests based on the *IDH1*1 gene for quantifying patulin-producing molds. When used in food, the newly developed RTi-PCR SYBR Green and TaqMan probes demonstrated significant sensitivity. Both RTi-PCR methods detected ten conidia per gram of food matrices, with an excellent linear relationship between the number of *IDH1* gene units and Ct values (Ngolong Ngea et al., 2020). As a result, real-time quantitative PCR (qPCR) has been shown to detect and quantify toxic molds in food before mycotoxin production” Similarly, Crespo-Sempere et al. (2013) developed a sensitive and efficient TaqMan qPCR multiplex method for quantifying fungi that produce mycotoxins and patulin. In order to generate PCR products specific to *B. nivea*, a PCR using conventional primer sets (B. nivea1F/1R) was completed (Kizis et al., 2021). Similarly, other amplification reactions using the standard primer sets B. lag 1F/1R, B. fulva 1F/1R, and B. zol3 F/R produced PCR products specific to *B. zollerniae*, *B. fulva*, *B. lagunculariae*, and *B. lagunculariae*, respectively. Early detection is essential for figuring out the crucial steps to get rid of moulds that produce patulin and, as a result, limit patulin in food. Early identification of patulin producers in food using PCR techniques will help stop patulin contamination after food production. Primers created from patulin biosynthesis genes are now readily available, and PCR and real-time PCR-based molecular techniques are evolving quickly. The existing primers, which were developed based on the search for patulin biosynthetic pathways in all potential patulin producers, not solely *P. expansum*, are not specific to all fungi that produce patulin.

### 5.3 Liquid-liquid extraction method

The extraction process is the initial stage in food analysis. It affects the target compound’s purity and concentration in the extracted sample. LLE is one of the most widely used methods for extracting compounds. It involves using two solvents that are either immiscible or partially miscible with each other to separate one or more species. This technique has been employed in the analysis of aqueous samples in various studies, including mycotoxin detection in different foods. For HPLC-UV analysis of apple juice and apple puree, the Association of Official Analytical Chemists (AOAC) suggests utilizing LLE with ethyl acetate, followed by cleanup using sodium carbonat. This method (MacDonald et al., 2000) was recently used to extract mycotoxin from apples and other fruit juices, with or without some modifications (Shephard and Leggott, 2000; Tamura et al., 2011; Malachová et al., 2014; Alshannaq and Yu, 2017; Iqbal et al., 2018). Unfortunately, because patulin is more stable in acidic media, adding sodium carbonate to the sample raises the pH and causes patulin degradation. The issue can be avoided by using different salts, like sodium sulfate and sodium hydrogen carbonate, as alternative SPE cleanup procedures (Liao et al., 2019). LLE has the disadvantage of requiring many organic solvents and taking a long time to prepare samples for analysis. To extract patulin from apple-based products like apple juice concentrate, mixed apple juice, and baby foods, the method involves using 25 mL of ethyl acetate to process 10 g of the sampled. After shaking vigorously for 3 minutes and centrifuging for 5 minutes, 20 mL of ethyl acetate is used to extract the aqueous phase twice. Sodium carbonate is added three times (in 2 mL portions) followed by shaking, and then one 5 mL portion of ethyl acetate is added.

The samples underwent chromatographic analysis after being processed through the following steps: pH adjustment, evaporation to dryness, reconstitution, and filtration (Sieper et al., 2019). Although this approach has shown to have substantial analyte recuperation rates in apple juice, achieving 92.0% and 98.0% recoveries for 10 µg kg^-1^ and 100 µg kg^-1^ of patulin spiking, respectively, it is widely recognized that it is a time-intensive method that demands a considerable amount of data. Moreover, the method generates a substantial amount of ethyl acetate waste (70 mL per sample), which can pose a danger when produced in large amounts (Yap, 2015). To extract patulin from a 10 g sample, it was diluted in 10 mL of water and subjected to three rounds of extraction using 50 mL, 25 mL, and 15 mL of ethyl acetate, respectively. After shaking for 10 minutes and centrifuging for 5 minutes, 9 mL of sodium carbonate solution was added, and the organic phase was agitated. The lower layer (sodium carbonate solution) was then extracted with 10 mL of ethyl acetate. The combined organic layer underwent dehydration, evaporation, reconstitution in a solvent, solid-phase extraction (SPE) purification, and filtration before chromatographic analysis. The method achieved satisfactory patulin recoveries (with a mean of 95%) when tested on baby food matrices spiked with mycotoxin at concentrations of 5-20 µg L^-1^. However, the drawbacks of this technique include the use of a large amount of ethyl acetate per sample (100 mL) and the extended time required for sample preparation before chromatographic analysis. Recent developments in LLE have introduced an intriguing alternative to traditional LLE methods. Ultrasonic techniques have emerged as a promising alternative for pre-treating juices and other fruit matrices for patulin determination using LLE (Gavahian et al., 2020). This method involves utilizing a blend of two solvents that do not mix with each other, namely the water present in the fruit matrix and an organic solvent. The approach enables the extraction of several samples simultaneously, while minimizing the quantity of material and organic solvent required (Basheer et al., 2010). The successful extraction of patulin from apple juice was achieved by utilizing ethyl acetate, while from whole apples, an ammonium acetate-acetic acid solution in a methanol-water solution (95:5, v/v) was used (Ohta et al., 2017). Vortex-assisted liquid-liquid microextraction (VALLME) is another technique that relies on lLLE. In this method, a small amount of extraction solvent is mixed into an aqueous sample using vortex agitation, creating tiny droplets that enhance the extraction of target compounds by minimizing the diffusion distance and increasing the surface area available for extraction (Yiantzi et al., 2010). After the phases have been separated, the organic layer can be collected and used directly in the chromatography system (**Table 4**).

**Table 4:** *Alternaria* mycotoxin detection using LC-UV/DAD/FLD/ECD/ELSD.

Alternaria	Sample	Analytical Technique	Clean-Up/Extraction	LODs	References
AOH	Tomato Pulp	HPLC-DAD	Methanol	5.0 μg kg^-1^	da Motta and Valente Soares, 2001
AME	–	–	–	–	–
TeA	–	–	–	–	–
ATX-I	Tomatoes	HPLC-ECD	Aqueous ammonium sulphate and methanol	Subparts/million	Visconti et al., 1991
ATX-II	–	–	–	–	–
AOH	Tomato Paste	HPLC-FLD SPE	QuEChERS	1.93ug/L^-1^	Fente et al., 1998
AOH	Pomegranate Fruit and Juice	HPLC-DAD	SPME	15–20 μg kg^-1^	–
AME	–	–	–	–	–
TeA	–	–	–	–	Myresiotis et al., 2015
TeA	Wine	LC-DAD	SPE	70 μg L^-^	Abramson et al., 2007; Myresiotis et al., 2015
AOH	Tomato and Products	LC-UV	SPE	5 ug/kg^-1^	–
AME	–	–	–	10 ug/kg^-^	–
TeA	–	–	–	20 ug/kg^-1^	Solfrizzo et al., 2004
AME	Tomato and Products	LC-UV	SPE	3 ug/kg^-1^	Stack et al., 1985
TeA	–	–	–	20 ug/kg^-1^	–
AOH	–	–	–	0.8-0.4 ng/ml	–
	–	–	–	0.5-0.4 ng/ml	–
AME	wine and juice	LC-UV	SPE	39 ng/ml	–
	Red grape	LC-UV	SPE	0.27 ng/ml	–
	White wine	–	–	–	Broggi et al., 2013

### 5.4 Solid-phase extraction method

Solid-phase extraction (SPE) is another environmentally-friendly approach in chromatographic analysis sample preparation. SPE techniques have become increasingly popular due to their several advantages over the conventional LLE methods, such as the requirement of a reduced number of samples and organic solvents for analysis, elevated recovery rates, and shorter processing durations (Kole et al., 2011). SPE works by dissolving or suspending samples in solvents (such as acetonitrile) and allowing them to pass through a solid phase. SPE works by dissolving or suspending samples in solvents (such as acetonitrile) and allowing them to pass through a solid phase separating analytes according to how well they adhere to the sorbent. This method makes it possible to isolate, concentrate, and purify the target molecule. in this investigation of patulin contamination in fruit-based commodities such as juices, purees, and jams, researchers recognized the value of SPE and implemented it individually (Vandendriessche et al., 2013; Seo et al., 2015; Arghavani-Beydokhti et al., 2018).

Nevertheless, as with every other methodology, SPE also has certain boundaries. The utilization of clarified juice, dried fruit, and other fruit-based products with intricate compositions was deemed unsuitable (Ji et al., 2017). SPE columns such as MycoSep 228 are commercially available and have been specifically designed for the detection of patulin in food. In this study, the effectiveness of the MycoSep 228 column was assessed for identifying patulin in homemade apple and hawthorn beverages (Seton et al., 2012). New sorbents have been created as an alternative to commercially available SPE columns to improve the effectiveness of separating patulin from other components of the fruit matrix. The removal and pre-concentration of patulin from fruit products have been studied using a variety of polymers as solid-phase sorbents (Anfossi et al., 2010; Zhou et al., 2019).

### 5.5 Molecularly imprinted polymers extraction

Molecularly imprinted polymers (MIPs) were created utilizing the oxindole molecule as a template to selectively bind mycotoxins in apple juice (Members et al., 2012). Methacrylic acid, which serves as the functional monomer, and ethylene glycol dimethacrylate were used to create the polymer *via* non-covalent free radical polymerization (cross-linker). Regrettably, the proposed method is overly complicated to prepare MIPs, and the recovery gains are only marginal (84.31 to 88.89%) was observed. Zho and colleagues proposed a radical polymerization method called Grafting as an alternative approach to MIPs (Members et al., 2012; Huen and Daoud, 2017; Zhou et al., 2019), In the production of a patulin-selective SPE sorbent, a silica surface was initially pre-grafted with amino groups, and MIP was created in the presence of acrylamide (functional monomer), ethylene glycol dimethacrylate (cross-linker), and 6-hydroxynicotinic acid (a template substitute) in a series of steps. The ultimate material was successfully employed to analyze patulin, with the analyte recoveries ranging from 90.08 to 96%(Sadok et al., 2019a)

### 5.6 QuECHERS, and safe extraction method

The QuEChERS approach, which stands for quick, easy, cheap, effective, rugged, and safe, has gained popularity as a pre-treatment method for chromatographic analysis due to its many benefits. This method typically involves the extraction of a 10 g homogenized sample in 10 mL acetonitrile, followed by the addition of a salt mixture (4 g magnesium sulfate anhydrous and 1 g sodium chloride) and separation of the extract. Then, a dispersive solid-phase extraction (DSPE) is performed to purify the 1 mL acetonitrile extract using 150 mg anhydrous magnesium sulfate and 25 mg PSA sorbent (Zhao et al., 2012). Dispersive solid-phase extraction (dSPE) has several advantages over traditional SPE. It eliminates the need for conditioning and elution steps, reduces sorbent consumption, and eliminates the need for additional equipment such as vacuum/pressure or flow control devices. In dSPE cleaning, sorbents are chosen to selectively retain interfering compounds extracted from the sample matrix, while retaining the analytes in the liquid phase. Several dSPE sorbents have been evaluated to ensure satisfactory recoveries and precise results. For example, the PSA sorbent effectively removes various polar matrix components found in food extracts, including organic acids, polar pigments, and sugars. Carbon-based sorbents are suitable for removing carotenoids, chlorophyll, and sterols, while octadecyl silica (C_18_) is recommended for samples with high fat and wax content (Sugitate et al., 2012). The original QuECHERS procedure has since been modified to suit specific applications better. Instead of acetonitrile, the extraction solvents and salt mixture composition used in the separation and purification steps were optimized for patulin quantification in fruit matrices (Dzuman et al., 2015) used methanol, a water-acetonitrile mixture (Sirotkin and Kuchierskaya, 2017), or acetonitrile that has been acidified with acetic acid (Burin et al., 2011). To extract patulin, it is possible to utilize the aforementioned method. In order to improve pH control and increase recovery rates, it may be necessary to add buffering salts like sodium citrate and sodium hydrogen citrate sesquihydrate to the standard sodium chloride and magnesium sulfate anhydrous mixture. This modification can improve the separation of matrix compounds (Fernandes et al., 2011; Sadok et al., 2018). The purification step was carried out by (Dušek et al., 2018) using dSPE and only in rare cases by traditional SPE columns (Han et al., 2012). Although the QuEChERS methodology offers several benefits, it utilizes a relatively high amount of extraction solvents in comparison to the DLLME and VALLME techniques mentioned earlier.

Furthermore, the type and number of salts used in the salting-out step affect extraction efficiency significantly. To prevent the extraction of unwanted compounds (such as sugars and pigments) from the sample matrix, it is necessary to perform an optimization step to identify the most effective conditions (Abu-Alsoud and Bottaro, 2021). In addition, post-extraction cleaning is typically required with the QuEChERS approach, which can lead to increased preparation time and analysis costs. The multi-step nature of this method can also increase the risk of target compound loss, which can be mitigated by incorporating an internal standard early in the sample preparation process. However, this can introduce another variable that must be accounted for during method development.

### 5.7 Matrix solid-phase dispersion extraction method

Matrix solid-phase dispersion (MSPD) is an alternative method worth considering for preparing solid and semi-solid samples for chromatographic analysis. This approach involves mechanically mixing the sample with a solid support (typically C_18_ or C_8_) to create a single stationary phase, which is then placed into a disposable cartridge. The desired compounds are eluted using an appropriate solvent, and the resulting eluent can then be further processed (through evaporation, reconstruction, and analysis). However, the MSPD approach can be time-consuming when targeting specific substances, and it requires relatively large amounts of organic solvents for analyte extraction (typically 10-15 mL per sample), as well as careful attention to sample/dispersant mixing and column packing material selection. Before eluting a sample, a column may occasionally need to be washed, as in SPE. This extra step increases the number of organic solvents used and lengthens the sample preparation process (Hasinger et al., 2018). The extraction of patulin from rot apples and apple juice concentrate was tested using the MSPD methodology. The MSPD methodology extracted patulin from rotten apples and apple juice concentrates (Li et al., 2017b).

### 5.8 Chromatography methods

The most commonly used chromatographic technique for determining patulin in various fruits and their products is HPLC coupled with either ultraviolet (UV) or diode array (DAD) detectors. In HPLC separations, a mobile phase typically consisting of water and a small amount of acetonitrile (5-10%, v/v) is used. To ensure patulin stability in the mobile phase, chloric acid is often added to acidify it, since patulin tends to be more stable in acidic environments (Berthiller et al., 2017; Sadok et al., 2018). This spectrum is used for patulin identification and quantification because the molecule’s maximum absorbance is at 276 nm (Piletska et al., 2017; Iqbal et al., 2018; Zhao et al., 2019; Fu et al., 2020). Detection based on this wavelength suffers from low selectivity due to interference from phenolic compounds, especially 5-hydroxymethylfurfural (5-HMF), which is formed during the thermal treatment of food as an intermediate product of acid-catalyzed hexose degradation and the decomposition of 3-deoxyosone in the Maillard reaction (Shapla et al., 2018). Several studies have shown both positive and negative effects of 5-HMF on human and animal health, including its mutagenicity (Perera et al., 2012). 5-HMF is almost undetectable in fresh fruits but abundant in processed foods. Therefore, the presence of 5-HMF may be useful in evaluating thermal damage or aging in food products. Additionally, 5-HMF is the predominant contaminant found in apple-derived samples during mycotoxin analysis (Al-Hazmi, 2010). Both patulin and 5-HMF absorb UV light strongly, have similar retention times under chromatographic conditions, and thus tend to peak overlap. Many HPLC-UV studies took on the analytical challenge of improving patulin and 5-HMF peak separation. Optimal analytical conditions for the analysis of apples and apple-based products have been established (Li et al., 2017c).

Liquid chromatography-mass spectrometry (LC-MS) is a popular tool for food analysis because of its adaptability, specificity, and selectivity. Most studies that focus on patulin monitoring in fruits use LC coupled with triple tandem quadrupole mass spectrometry (LC-QQQ) in negative-ion mode. The patulin precursor ion is typically chosen as m/z 153, which corresponds to a patulin molecule after losing a proton [M-H]. The mobile phase is composed of acetic acid and methanol or acetonitrile (Madani-Tonekaboni et al., 2015). Alternatively, this mycotoxin analysis employs ammonium acetate (Panwalkar et al., 2017; Rodriguez et al., 2021). The target molecule is highly polar, which results in low sensitivity for the LC-MS analysis. The problems can be attributed to the inadequate ionization of patulin under source and atmospheric pressure chemical ionization (APCI) conditions (Nielsen and Larsen, 2015). LC-MS analysis of complex matrices like fruit samples must consider matrix effects that can lead to signal suppression or enhancement of the target molecule. To reduce these effects when quantifying patulin, using an isotopically labeled patulin standard and a matrix-matched methodology is recommended. However, most LC-MS protocols are designed for multi-target analyses, which can reduce sensitivity and may not meet the EU’s legal restrictions for patulin quantification in baby food. For example, LC-QQQ has been used to identify 33 pesticides or degradation products in addition to patulin in apples, and the method was validated using four food matrices. In the apple matrix, the LOQ for patulin was 119.7 µg kg-1, with a 77% recovery rate. Patulin had a LOQs of 5 µg L-1 and a 78% recovery rate (Nakatsuji et al., 2015). The patulin levels in various fruit juices (apple, grape, orange, blueberry, lemon, pear, mango, and coconut) and dried fruits (apple, apricot, kiwi, prune, pineapple, papaya, mango, fig) were assessed, with estimated recoveries exceeding 92% for the apple juice matrix. Moreover, utilizing the QuEChERS method for sample preparation of apple and pear-based products prior to LC-MS/MS analysis resulted in higher recoveries ranging from 94% to 104%, with LOQs of up to 10 µg/L for all matrices tested (El-Ramady et al., 2015). The advanced LC method with a triple quadrupole analyzer was used to determine individual patulin levels in four different apple matrices, including juice, fruit, puree, and compote (El-Ramady et al., 2015). The sentence describes the results of a study that used LC-MS/MS method to measure patulin levels in various fresh fruits and their products. The study found that LOQs for all matrices ranged from 2 to 15 µg kg-1, and the average recoveries ranged from 71 to 108 per cent. The fruits and products tested included apples, pears, apricots, peaches, bananas, grapes, plums, strawberries, raspberries, blueberries, blackberries, sour cherries, as well as their juices and pulps. The study aimed to track the levels of patulin in these fruits and products to ensure food safety (Azari et al., 2020). The method using LC-MS/MS has been able to detect patulin levels with LOQs ranging from 0.8 to 2.4 µg kg-1, and it has been applied to the analysis of patulin content in apples and apple-based products. However, it should be noted that this method may not be suitable for the analysis of patulin in complex matrices such as dried or pigmented fruits (such as berries and sour cherries) and their derived products, where LLE-based methods may not be effective It requires changing current analytical protocols or creating new ones, such as more complex sample preparation. Patulin quantification is challenging at the legislative level due to various sample components that adversely affect chromatographic peak resolution and ionization efficiency and lead to unsatisfactory recovery. Recent advances in this field have primarily focused on SPE and QuEChERS adjustment. It enabled the development of efficient protocols for removing interfering compounds (such as phenolic molecules) from complex fruit matrices. Several modifications to the original QuEChERS protocol, including using MIPs as SPE sorbents, have been proposed to enhance sample cleaning and patulin pre-concentration. Although it is still preferred, the HPLC-UV method has a lengthy analysis time for mycotoxin analysis in fruit commodities. (This is required for good peak resolution) and, as a result, relatively high mobile phase consumption. Because of this, mass spectrometry detection and UHPLC systems have surpassed the HPLC-UV method in analyzing organic compounds, which is now being phased out. Because of poor ionization in a mass spectrometer source, patulin is frequently omitted from a list of multi-target mycotoxin analyses. Instead, it is determined on an individual basis through a separate protocol. It appears to be a significant barrier to developing multi-mycotoxin approaches, so resolving the patulin sensitivity issue on the future path of LC-MS-based methods will require careful attention.

Tin layer chromatography has been employed as a rapid and direct qualitative analysis technique in various fields, including the determination of *Alternaria* mycotoxin levels. In a study using a solvent system of chloroform/acetone (97:3, v: v) and TLC-UV, *Alternaria* mycotoxins (AME, AOH, TeA, ATX-I, and ATX-II) were detected in tomatoes. The predominant mycotoxins found in spoiled tomatoes were AOH, AME, and TeA, with LODs of 100, 100, and 700 µg kg^-1^ for AOH, AME, and TeA, respectively (Gašparović et al., 2015). The presence of AOH, AME, ALT, ATX-I, and TEN in *A. alternata* IMI 354942 cultures was detected using TLC-UV. The LODs for AOH, AME, ALT, ATX-I, and TEN were 250, 125, 250, and 250 µg L^-1^, respectively. Compared to high-performance thin-layer chromatography (HPTLC), TLC showed superior separation efficiency and detection sensitivity (Gökbulut, 2021). The study detected AOH and AME mycotoxins in raspberry, tomato, wheat, and oat samples using HPTLC and densitometry techniques, with a LODs of approximately 60 µg kg^-1^. The same HPTLC method was also used to quantify AOH, AME, ALT, and TeA mycotoxins in fresh grape juice, must, and wine, with LOQs of 1.5 µg L^-1^ for AOH and AME, and 7.5 µg L^-1^ for TeA (Liang et al., 2021). Although TLC has lower separation efficiency and detection sensitivity compared to HPLC and GC, it is still an essential tool for mycotoxin detection in various matrices due to several advantages, such as ease of operation, rapidity, cost-effectiveness, and the use of less solvent than LC. Additionally, TLC has no memory effects, making it a more environmentally friendly optio” (Zhang et al., 2021). Furthermore, the detection sensitivity of HPTLC is higher than that of GC and HPLC with a densitometric detector.

In recent years, reversed-phase LC coupled with various classical detectors such as ultraviolet detector (UVD), diode-array detector (DAD), fluorescence detector (FLD), electrochemical detector (ECD), evaporative light-scattering detector (ELSD), and mass spectrometry (MS) has become the preferred method for detecting *Alternaria* mycotoxins, supplanting GC and TLC. Among these detectors, LC-UV is a popular method due to the ability of most organic and inorganic molecules to absorb ultraviolet light. To detect *Alternaria* mycotoxins in carrots, researchers utilized SPE as a pre-treatment step and employed reversed-phase LC with a UV diode array detector (LC-UV/DAD). The study found that LODs for TeA, ATX-I, AME, and AOH were 20, 20, 10, and 5 µg kg^-1^, respectively (Gotthardt et al., 2019). Canadian ice wines contain TeA, while Estonian grain wines contain LC-DAD AOH (Asam et al., 2012). The LODs for the analytes were determined using LC-DAD, and were found to be 100 µg kg^-1^ and 70 µg L^-1^ for one or more of the *Alternaria* mycotoxins. In the case of TeA, it is known to act as an effective chelating agent, forming complexes with metal ions. Therefore, when detecting TeA, zinc sulfate (ZnSO_4_) is often added to the mobile phase to enhance the sensitivity of the detection method (Santos et al., 2012), Due to its low sensitivity, LC-DAD is less frequently used than UV detectors to detect *Alternaria* mycotoxins. Contrarily, the LC-UV/DAD method of diode array UV detection has a high sensitivity (De Vargas et al., 2015). A new sampling technique for detecting pesticides in fruits and vegetables has been developed, which is quick, simple, and efficient. QuEChERS, an acronym for quick, easy, cheap, effective, rugged, and safe, is an additional method to SPE and SPME techniques (Lingwal et al., 2022). By employing HPLC-DAD in combination with the QuEChERS extraction method, the levels of AOH, AME, and TEN were measured in both pomegranate fruit and juice, with LODs falling between 15 to 20 µg kg^-1^, and the LOQs ranging from 50 to 66 µg kg^-1^ (Petsas and Vagi, 2020). HPLC-FLD is an abbreviation for the FLD acronym, which is frequently used in high-performance liquid chromatography. Compared to UV and DAD detectors, it offers greater selectivity and sensitivity, with a LOD of up to µg L^-1^. The quantity of AOH present in tomato paste was evaluated using HPLC-FLD, following extraction of the sample through SPE cartridges. The LOD of AOH was found to be 1.93 µg L^-1^, with a linear range of 5.2-196 µg L^-1^ (Zhang et al., 2020a). Due to the absence of fluorescent functional groups in TeA, its detection is not feasible with the FLD detector. Consequently, LC-UV and LC-DAD methods are more commonly employed than LC-FLD for the identification of *Alternaria* mycotoxins, as they can detect TeA as well. LC-ECD, a popular technique for analyzing trace samples, has a sensitivity of 106 µg L^-1^, and is exclusively used to detect electroactive molecules that can be readily oxidized or reduced. AOH, AME, ATX-I, and ATX-II, all being electroactive, are among the *Alternaria* mycotoxins that can be detected using LC-ECD (Man et al., 2017). To enhance the detection sensitivity of ATX, a dual in-series electrode system can be coupled with an HPLC method in the “redox” mode (Sontag et al., 2019). Samples containing ATX-I and ATX-II were extracted from maize, rice, and tomatoes that were infected, and LOD levels achieved were below one part per million (sub-ppm) (Sontag et al., 2019). The detection of AAL toxin has traditionally depended on time-consuming derivatization or immunoassay methods since it lacks a UV chromophore, which is why ELSD-LC has been used as an alternative (Lagarde et al., 2018). A sensitive and rapid analytical method was developed for the quantitative detection of AAL toxins in fungal culture, which involves coupling a C_18_ reverse phase HPLC to an ELSD, with a LOD of around 6000 µg L^-1^. However, this ELSD method emits harmful exhaust gas during the detection process and requires a signal transducer in conjunction with LC and high-pressure nitrogen or air configuration. In recent years, MS detection techniques, especially LC-MS/MS or LC-MSN interfaces based on ESI or APCI, have outperformed FLD, UV, DAD, and ECD detectors in the simultaneous detection and quantification of *Alternaria* mycotoxins in various samples without derivatization. The ESI source has been the most commonly used for detecting *Alternaria* mycotoxins due to its higher sensitivity than APCI. Using LC-MS analytical methods, dibenzopyrone derivatives Toxins AOH, AME, and ALT can be detected. A pretreatment method utilizing SPE was employed for the detection of AOH and AME in flavedo, using HPLC-MS/MS. The results indicated a linear range of 0.50 -20.0 µg kg^-1^, with a LOD and LOQ lower than 0.13 and 0.50 µg kg^-1^, correspondingly, and an RSD of 114.4%. Subsequently, a HPLC-MS/MS method was developed to detect 23 mycotoxins in 6 food supplements (Myresiotis et al., 2015). The sample was initially obtained using a mixture of ethyl acetate and formic acid (95:5 v/v), followed by purification using an OASIS HLBTM SPE column. of the 23 mycotoxins, the LODs for ALT, AOH, and AME were 2, 8, and 30 µg kg^-1^, respectively. The LOQs for these mycotoxins were approximately three times higher than their respective LODs. Furthermore, although the sample preparation differed, HPLC-MS/MS detected 23 mycotoxins in sweet pepper (Rico-Yuste et al., 2018). The multi-mycotoxin LC-MS/MS method that was developed successfully fulfilled the method performance criteria that were specified in Commission Regulation (EC) no. 401/2006 (Matumba et al., 2015). The QuEChERS extraction method can be employed in conjunction with LC-DAD and LC-MS techniques to simultaneously detect *Alternaria* mycotoxins. AOH and AME, both of which are multi-mycotoxins, were extracted utilizing QuEChERS and subsequently identified through LC-ESI-MS/MS. The LODs for AOH and AME were 10 and 6g kg^-1^, respectively (Zervou et al., 2017). UHPLC (ultra-high-performance liquid chromatography), has been extensively utilized in the analysis of mycotoxins, as well as in the LC and HPLC methods mentioned earlier. For example, the QuEChERS method was utilized to extract AOH, AME, TEN, ALT, and ATX-I from tomato products, fruit juices, and vegetable juices, with detection being carried out through UPLC-MS/MS. The findings indicated that LODs, LOQs, and LOQs of these toxins in tomato products, fruit juices, and juices ranged from 3.0-8.3, 9.8-61.5, and 1.1-5.7 µg kg^-1^, respectively (Smoluch et al., 2016). Regenerate Performed detection of *Alternaria* mycotoxins with different chemical structures at the same time successfully used HPLC-ESI-MS/MS in foods from German markets to quantify the nine *Alternaria* toxins (AOH, AME, TeA, ALT, ALT, TEN, and ATX-I). The LODs and LOQs were determined to be 2.8-5.4 and 9.3-8 µg kg^-1^, respectively, while for TA 2, the values were found to be 1.2-7 and 3.8-55 µg kg^-1^. LC-APCI-MS/MS was used to identify five *Alternaria* mycotoxins, including AOH, AME, TeA, ALT, and TEN, in various food products such as apple juice, beers, tomato products, olives, and dried basil, with LODs and LOQs ranging from 0.16-2.31 µg kg^-1^ and 0.54-41.04 µg kg^-1^. AOH was found to be the most common *Alternaria* mycotoxin, followed by ALT. Utilized UPLC-ESI-MS/MS to quantify the levels of these mycotoxins in tomato products, and TeA was found to be the most frequently detected toxin, with a maximum concentration of 790 µg kg^-1^ in 81 out of 84 samples (Lawal et al., 2019). On the other hand, AOH and AME were found to have lower concentrations, ranging from 1 to 34 µg kg^-1^ and 5 to 9 µg kg^-1^, respectively. Therefore, the results suggest that TeA is the most common *Alternaria* mycotoxin (Noser et al., 2011). LC-ESI-MS/MS analysis detected AOH, AME, ATX-I, and TEN mycotoxins in various samples, in addition to other mycotoxins (Puntscher et al., 2018; Gotthardt et al., 2019). (**Tables 5** , **6**).

**Table 5:** *Alternaria* mycotoxin detection using LC–MS.

Alternaria	Sample	Analytical Technique	Extraction/Clean-Up	Limit of Detection (LODs) (µg/kg)	(LOQs : Limit of quantitation) (µg/kg)	References
AOH	Wines, grapes juices	LC-ESI-MS/MS	SPE	0.01-0.8	–	–
AME	and cranberry juice	–	–	–	–	Juan et al., 2017
AOH	Tomato, wine, apple	LC-ES-MS/MS	Water-formic acid acetonitrile	–	1.5-5.0	–
AME	apple juices	–	(84/16/1, v/v/v)	–	–	–
ALT	–	–	–	–	–	López et al., 2016
AOH	FRUITS	UPLC/ESI-MS/MS	Acetonitrile/water/acetic 79:20:1, v/v/v	2	–	–
AME	–	–	–	0.1	–	–
ALT	–	–	–	6	–	–
TEN	–	–	–	0.5	–	Haque et al., 2020
AOH	Beer	UHPLC-orbitrap MS	Acetonitrile	–	–	–
AME	–	–	–	–	–	–
ALT	–	–	–	–	–	Tittlemier et al., 2019
AOH	tomato products	UPLC–MS/MS	QuEChERS	3.1-18.3	9.8-61.5	–
AME	Fruits	–	–	–	1.1-5.7	Walravens et al., 2016
AOH	fruits such as apple	SPE	–	–	44563	–
AME	orange, sweet cherry	–	–	–	–	–
ALT	and tomato	–	–	–	–	Wang et al., 2021
AOH	tomato products	UPLC-ESI-MS/MS	SPE	4	–	–
AME	–	–	–	1	–	–
ALT	–	–	–	2	–	–
TeA	–	–	–	2	–	–
ATX-1	–	–	–	–	–	–
TEN	–	–	–	2	–	Noser et al., 2011
AOH	dried fruits	LC–MS/MS ESI	Acetonitrile/water/acetic acid, 79:20:1, v/v/v	5	–	–
AME	–	–	–	8	–	–
ATX-1	–	–	–	3	–	–
TEN	–	–	–	0.4	–	Warth et al., 2012
AOH	apple Juices, beers, tomatoes, olives	LC-APCI-MS/M	SPE	0.16-12.31	0.54-41.04	Garganese et al., 2016
AOH	tomato and fruit juices	HPLC-ESI-MS/MS	Methanol/water/formic acid (49:50:1, v/v/v)	0.04–0.4	0.6–9.3	–
AME	–	–	–	0.8–24 2.5–81	0.1–1.2	–
ALT	–	–	–	1.3–19	4.4–62	Hickert et al., 2016
TeA	–	–	–	–	–	–
TeA	Olive, olive oil, olive husks	–	–	–	100 μg kg^-1^	Nunes et al., 2011
ATX-1	–	–	–	–	200 μg kg^-1^	–
ALT	–	–	–	–	100 μg kg^-1^	–
AME	–	–	–	–	30 μg kg^-1^	–
AOH	–	–	–	–	50μg kg^-1^	–
AOH	tomatoes	TLC-UV	–	–	100 μg kg^-1^	–
AME	–	–	–	–	100 μg kg^-1^	–
TeA	–	–	–	–	700 μg kg^-1^	–
AOH	Fruit and vegetable Products	TLC-UV	–	–	3 μg kg^-1^	Gagic et al., 2018
AOH	Raspberries, tomatoes	HPTLC-densitometry	–	–	–	–
AOH	Grape juice, and wine	HPTLC	–	–	60 μg kg^-1^	Matysik and Giryn, 1996
AME	–	–	–	–	1.5 μg L^-1^	
TeA	–	–	–	–	7.5 μg L^-1^	Berthiller et al., 2017

**Table 6:** *Alternaria* mycotoxin detection using LC-MS and SIDA.

Mycotoxin	Sample	Analytical Techniques	Clean-up/extraction	LOQs	LODs	Reference
TEN	Juice	SIDA, LC-MS/MS	C_18_-phenyl SPE	–	0.10-0.99 ug/kg^-1^	Liu and Rychlik, 2013
TeA	Tomato	HPLC-MS/MS, SIDA	RP18-SPE	2.89 μg kg^-1^	0.86 μg kg^-1^	Lohrey et al., 2013
TeA	Beverages	SIDA, LC-MS/MS	C_18-_SPE	–	–	Asam and Rychlik, 2013
TeA	fruit juices	SIDA, LC-MS/MS	C_18-_SPE	0.5 μg kg^-0^	0.15 μg kg^-1^	Berthiller et al., 2018
TeA	Tomato	SIDA, LC-MS/MS	–	0.3 μg kg^-1^	0.1 μg kg^-1^	Asam et al., 2011
AME	fruit products	–	–	0.1–5 μg kg^-1^	–	Asam and Rychlik, 2015
AOH	fruits,	SIDA, HPLC-MS/MS	RP18-SPE	–	13–250 μg kg^-1^	Ma et al., 2019
AOH	juice	SIDA, HPLC-MS/MS	RP18-SPE	0.09 μg kg^-1^	0.03 μg kg^-1^	Man et al., 2017
AME	Apple Juice	UPLC-MS/MS	C_18_	–	2.20–3.10ug/L	–
TeA	Apple Juice	UPLC-MS/MS	C_18_	–	11.90–20.60	–
AME	Apple Jam	UPLC-MS/MS	C_17_	–	3.36-4.42ug/kg^-1^	–
	Apple Vinegar	UPLC-MS/MS	C_18_	–	15.50 ug/kg^-1^	Quan et al., 2020

### 5.9 Gas chromatography method

*Alternaria* mycotoxins were also used with various detection techniques to detect GC has high sensitivity and selectivity, especially GC-MS, and can detect some substances in the blend. Despite being small, non-volatile, and polar, many *Alternaria* mycotoxins can still be detected using methods that are sensitive to non- and half-polar, volatile, and semi-volatile compounds (Anfossi et al., 2016). Thus *Alternaria* mycotoxins usually need to be derived before the analysis of GC-MS (Anfossi et al., 2016). TeA was derivatized by a compound of acetyl-trimethyl silane (6:2:9, v: v:v) and a mixture of acetylated ionization silane, trimethylated silane, and pyridine; TeA’s LOD was 100 µg kg^-1^, using GC and fire ionization sensor.

The researchers employed a two-step process of derivatization followed by GC-MS analysis using heptafluorobutyrate (HFB) and Trimethylsilyl (TMS) to detect mycotoxins such as AOH, AME, ALT, ALTX-I, and TeA (Amatatongchai et al., 2019). The results showed that GC separation of the *Alternaria* mycotoxin before MS detection is sufficient for both the HFB and TMS derivatives. The results showed that GC separation of the *Alternaria* mycotoxin before MS detection is sufficient for both the HFB and TMS derivatives. Apple juice contained AOH and AME LODs of 1 µg kg^-1^. Despite their high sensitivity, the GC-MS methods described above have not been widely used for detecting *Alternaria* mycotoxins. The main reason for this is that most *Alternaria* mycotoxins samples require derivatization in GC-MS detection process has certain limitations such as matrix interference, inadequate repeatability, high time consumption, costly derivatization reagents, and complicated operational procedures. Moreover, the technique can experience memory effects due to earlier sample injections. As a result of the time-consuming derivatization reactions required, the use of GC to determine *Alternaria* mycotoxins is limited.

### 5.10 SIDA method

Quantitative results must be corrected using special methods and suitable internal standards to consider the significant ion suppression that LC-MS experiences (Al-Lami et al, 2019). Several sample preparation and separation techniques are required because *Alternaria* can infest various analytical materials. SIDA (stable isotops dilution assays) is a fantastic method for recouping analyte losses during sample preparation and minimizing ion suppression at the ESI interface (Pavicich et al., 2020). Furthermore, SIDA is a useful tool in analytical applications, such as trace analysis, increasing the accuracy of quantitative results and improving the specificity of the determination (Graybill and Bailey, 2016). Thus far, the primary applications of SIDA for mycotoxin analysis have received critical attention (Malachová et al., 2018). HPLC-MS/MS was used to detect AOH and AME in fruit juices, and the reproducibility of the method was tested using spiked apple juice. The results showed that the reproducibility for AOH and AME was 100.5 ± 3.4% and 107.3 ± 1.6%, respectively. The LODs for AOH and AME were 0.03 and 0.01 µg/kg, and the LOQs were 0.09 and 0.03 µg/kg, respectively (Asam and Rychlik, 2015). Internal standards include [^13^C_20_]-ATXs, [^13^C_15_] AOH, and [^13^C_14_]-AME. In a stable isotope dilution, alternative labeling of [^13^C_20_]-ATXs, [^13^C_14_]-AOH, and [^13^C_15_]-AME were used as internal standards. The study found that samples contaminated with ATX may also contain other *Alternaria* toxins like AOH, AME, and TEN, but not necessarily in the same sequence(Liu and Rychlik, 2015). Additionally, the recoveries showed a range of 96 to 109%, while the inter- and intra-day RSDs were below 13%. Furthermore, the LODs and LOQs for AOH, AME, ATX-I, and ATX-II were determined to be 0.36 and 1.1 µg kg^-1^, 0.09 and 0.27 µg kg^-1^, 0.36 and 1.1 µg kg^-1^, and 0.53 and 1.6 µg kg^-1^, respectively [279, 280] is an internal standard. After acetoacetylation with diketene, the stable-isotope-labeled [^13^C_6_^15^N] To quantify TeA in tomatoes using LC-MS/MS, [^13^C_6_, ^15^N] TeA was synthesized by Dieckmann intramolecular cyclization and used as an internal standard. The LOD and LOQ for TeA were determined to be 0.1µg kg^-1^ and 0.3 µg kg^-1^, respectively. The TeA content in various foods was determined using SIDA, with LODs of 0.15 µg kg^-1^for fruit juices, 1.0 g kg^-1^ for cereals, and 17 µg kg^-1^ for spices (Pulkrabová et al., 2013). The study also investigated the TeA levels in infant foods and beverages. Popkin reported that the median TeA content in infant tea infusions was 2 µg L^-1^, while fennel tea infusions had TeA levels as high as 20 µg L^-1^. Moreover, the average TeA content in pureed food was found to be higher for tomatoes at 25 µg kg^-1^, and for bananas and cherries at 80 µg kg^-1^ (Asam and Rychlik, 2013) (**Table 7**).

**Table 7:** Country wise regulations on Maximum permitted limits (MPLs) of patulin and *Alternaria* content in fruits and their derived products.

Country	Commodity	Maximum Permitted Limits (MPLs)	References
China	Fruit products (with the exception of hawthorn sheet) Fruit and vegetable juice Liquor	50 μg kg^−1^	Li et al., 2017a
United States of America	Apple juice, apple juice concentrate, and apple juice component of a food that contains apple juice as an ingredient	50 μg kg^−1^	Cai et al., 2020
MERCOSUR member states	Fruit juice	50 μg kg^−1^	
Cuba	Fruits	50 μg kg^−1^	
European Union member states	Fruit juices, concentrated fruit juices as reconstituted and fruit nectars,	50 μg kg^−1^	Van Egmond and Jonker, 2008
	Spirit drinks, cider, and other fermented derived from apples or containing apple juice	50 μg kg^−1^	
	Solid apple products, including apple compote, apple puree intended for direct consumption (except baby foods)	25 μg kg^−1^	
	Apple juice and solid apple products, including apple compote and apple puree, for infants and young children	10 μg kg^−1^	
	Baby foods other than processed cereal-based foods for infants and young children	10 μg kg^−1^	
Iran	Fruit juices, nectarine, and fruit drinks	50 μg kg^−1^	Nascimento and Taniwaki, 2023
Israel	Apple juice	50 μg kg^−1^	
Japan	Apple juice	50 μg kg^−1^	
Republic of Korea	Apple juice, apple juice concentrate	50 μg kg^−1^	
Republic of Moldova	Juices, canned vegetables, fruits	50 μg kg^−1^	
Morocco	Apple juice (products)	50 μg kg^−1^	
Russian Federation	Apple, tomato, sea-buckthorn (canned)	50 μg kg^−1^	Dankvert, 2010
	Alcoholic free beverages, including juice containing and artificially mineralized drinks	50 μg kg^−1^	
	Vegetable, fruit juices, beverages, concentrates	50 μg kg^−1^	
	Semi-finished products (tomato pulp, apple pulp)	50 μg kg^−1^	
	James, marmalade, fruit pastes, comfitures, fruit and berries crushed with sugar and other fruit and berry concentrates with sugard	50 μg kg^−1^	
Singapore	Fruit Juices	50 μg kg^−1^	Lee et al., 2018
	Food for infants or young children (except processed cereal-based foods)	10 μg kg^−1^	
	Food containing fruit juice as ingredient	50 μg kg^−1^	
South Africa	All foodstuffs	50 μg kg^−1^	Van Egmond and Jonker, 2008
Switzerland	Fruit juices	50 μg kg^−1^	
Armenia	Tomato paste, apple	50μg kg^−1^	
Belarus	Mushrooms, fruits, vegetables	50 μg kg^−1^	
Ukraine	Vegetable and fruit-berry preserves and mixes for baby food.	20 μg kg^−1^	
	Vegetables, including potatoes, fruit and grapes, berries; vegetable, fruit, berry preserves in cans and jars	50 μg kg^−1^	
Canada	Fruits and fruit juices	50 μg kg^−1^	Canada, 2020
Australia New Zealand	Fruits, Apple juices, berries	50 μg kg^−1^	Sirisena et al., 2015
Brazil	Fruit and their products	50 μg kg^−1^	Brazil, 2001
India	Apple juices	50 μg kg^−1^	Shukla et al., 2014
Mexico	Fruit and juices	50 μg kg^−1^	Shukla et al., 2014
European Union	Tomato juices	30 µg/kg	C.R.N, 2006; Van Egmond and Jonker, 2008
Japan	Apple juices	30 µg/kg	Li and Beghin, 2014
China	Apple and tomato juices	50 µg/kg	

### 5.11 Enzyme-linked immunosorbent assay method

High-end equipment and highly trained personnel are required for instrumental analytical methods such as GC-MS and LC-MS. Despite the limitations of instrumental analytical methods, enzyme-linked immunosorbent assay (ELISA) has become a popular research focus for quantitative and semi-quantitative mycotoxin detection, including *Alternaria* toxin. ELISA stands out due to its simplicity, miniaturization, speed, and portability. It has been used to detect AAL, AOH, and TeA, and can be applied to determine the presence of *Alternaria* mycotoxin in foods. TeA was derivatized with succinic anhydride and then coupled with KLH to screen polyclonal antibodies. Competitive ELISA developed using these polyclonal antibodies showed higher sensitivity for TeA acetate than for TeA, with an average standard curve detection limit of 5.4 ± 2.0 µg L^-1^ for TeA acetate. The LODs for TeA in apples and tomatoes was found to be 25-50 µg kg^-1^(Gross et al., 2011; Wang et al., 2020).

## 6 Management of patulin and *Alternaria* mycotoxins in fruits

It is necessary to manage the presence of Patulin and *Alternaria* mycotoxins in fruits because these toxins can pose significant health risks to humans if consumed in large quantities. Patulin is a toxic secondary metabolite produced by several fungal species, including *Penicillium*, *Aspergillus*, and *Byssochlamys*, commonly found on fruits such as apples, pears, and grapes. Patulin has been linked to various health problems such as acute gastrointestinal distress, nausea, vomiting, and immune system suppression in animal studies, and possibly carcinogenicity in humans (Azam et al., 2021). On the other hand, *Alternaria* mycotoxins, including AOH, AME, and tentoxin, are also commonly found in fruits such as tomatoes, strawberries, and apples, and have been associated with several health problems such as genotoxicity, immunotoxicity, and carcinogenicity in animal studies, although their effect on humans is still unclear. Therefore, it is important to manage the levels of patulin and *Alternaria* mycotoxins in fruits to ensure that the fruits consumed by humans are safe and do not pose any health risks (El-Kady and Abdel-Wahhab, 2018). The management of patulin and *Alternaria* mycotoxins in fruits can be achieved through a combination of good agricultural practices, post-harvest handling, and processing to minimize its presence in fruits. The potential measures are pre-harvest management including proper crop management, including irrigation, fertilization, and pest control, can help reduce the occurrence of fungal infections in fruits. Use of resistant cultivars can also help reduce infection rates. Harvest management including proper harvesting techniques, including the use of sanitized equipment and the removal of infected fruits, can help minimize fungal contamination. Post-harvest handling including proper storage and transportation practices, temperature control and regular cleaning and sanitization, can help prevent fungal growth and mycotoxin production (Nan et al., 2022). Processing appropriate processing techniques, including washing, sorting, and disinfection, can help reduce the levels of mycotoxins in fruits and fruit products. And monitoring including regular monitoring of fruit and fruit products for mycotoxin levels can help identify potential contamination and allow for corrective action to be taken (Saleh and Goktepe, 2019). Certain management strategies including, physical methods, chemical methods, and biological method, to reduce the amount of patulin and *Alternaria* mycotoxins contamination in fruits are discussed below.

### 6.1 Physical methods

There are several physical methods that can be used to reduce the levels of patulin and *Alternaria* mycotoxins in fruits and their derived products. The most considerable physical methods to reduce these mycotoxins include; Thermal treatment (also known as heat treatment is one of the most common methods used to reduce mycotoxin levels in food products. High temperatures can denature or destroy the mycotoxins, but this method may also result in the loss of some nutrients and changes in the organoleptic properties of the food product) (Shanakhat et al., 2018); Irradiation (Irradiation is another method that can be used to reduce mycotoxin levels in food products. It involves exposing the food product to high-energy radiation, such as gamma rays, which can break down the mycotoxins. This method is effective in reducing mycotoxin levels but may also affect the sensory properties of the fruits and their derived products) (Akhila et al., 2021); Ultrasonic treatment (It is a non-thermal physical method that can be used to reduce mycotoxin levels in food products. This method involves exposing the food product to high-frequency sound waves, which can disrupt the mycotoxins’ structure and reduce their toxicity (Sipos et al., 2021). This method is effective in reducing mycotoxin levels while preserving the nutritional value and sensory properties of the food product); Cold plasma treatment (another non-thermal physical method that can be used to reduce mycotoxin levels in food products. This method involves exposing the food product to low-temperature plasma, which can break down the mycotoxins. This method is effective in reducing mycotoxin levels but may also affect the sensory properties of the food product) (Misra et al., 2019); and High-pressure processing (HPP) (HPP is a method that involves subjecting the food product to high pressure, which can destroy the mycotoxins. This method is effective in reducing mycotoxin levels while preserving the nutritional value and sensory properties of the food product) (Woldemariam and Emire, 2019). In conclusion, there are several physical methods that can be used to reduce mycotoxin levels in fruits and their derived products. These methods are effective in reducing mycotoxin levels while preserving the nutritional value and sensory properties of the food product. However, the choice of the most appropriate method will depend on the type of food product, the nature of the mycotoxin, and the desired level of reduction.

It worth to mention that, in the physical methods for the detection of *Alternaria* and patulin in fruits, high temperature treatments have been shown to be effective in controlling fungal growth and mycotoxin production. However, it can also cause damage to the fruit, reducing its quality and shelf life (Pal et al, 2017). Similarly, UV-C radiation has been reported to be effective in reducing *Alternaria* and patulin in fruits. However, it can also damage the fruit surface and lead to changes in color and texture. In contrast, cold plasma treatment has been shown to be effective in reducing *Alternaria* and patulin in fruits without causing any damage to the fruit surface. As it is a non-thermal process and does not affect the fruit quality and nutritional value (Khan et al., 2019). Moreover, MAP is also an effective method for controlling fungal growth and mycotoxin production in fruits. It involves modifying the atmosphere inside the package to slow down the ripening process and inhibit fungal growth. It is a non-invasive method and does not affect the fruit quality and nutritional value.

### 6.2 Chemical methods

There are several chemical methods that have been developed for patulin and *Alternaria* mycotoxin reduction in fruits. Some of the most commonly used ones include; Sodium bisulfite treatment (Sodium bisulfite is a chemical that can reduce the levels of patulin and *Alternaria* mycotoxins in fruits. The treatment involves soaking the fruits in a solution of sodium bisulfite for a specific period of time, which can vary depending on the type of fruit and the concentration of the solution); Hydrogen peroxide treatment (Hydrogen peroxide is a strong oxidizing agent that can be used to reduce the levels of patulin and *Alternaria* mycotoxins in fruits. The treatment involves spraying the fruits with a solution of hydrogen peroxide, which can effectively degrade the mycotoxins) (Paster and Barkai-Golan, 2008); Sodium hypochlorite treatment (Sodium hypochlorite is a chemical that is commonly used as a disinfectant. It can also be used to reduce the levels of patulin and *Alternaria* mycotoxins in fruits. The treatment involves washing the fruits with a solution of sodium hypochlorite, which can effectively remove the mycotoxins (Karlovsky et al., 2016b); Ozone treatment (Ozone is a powerful oxidizing agent that can be used to reduce the levels of patulin and *Alternaria* mycotoxins in fruits. The treatment involves exposing the fruits to ozone gas, which can effectively degrade the mycotoxins (Afsah-Hejri et al, 2020); and Ultraviolet treatment (Ultraviolet light can be used to reduce the levels of patulin and *Alternaria* mycotoxins in fruits. The treatment involves exposing the fruits to ultraviolet light for a specific period of time, which can effectively degrade the mycotoxins). It is important to note that while chemical methods can be effective in reducing the levels of mycotoxins in fruits, they can also have negative effects on the quality and nutritional value of the fruits (Akhila et al., 2021). Therefore, it is important to carefully consider the potential risks and benefits of using these methods before implementing them.

It is noteworthy that, in the chemical methods for the detection of *Alternaria* and patulin in fruits, these chemical methods require specialized equipment and trained personnel, making them more costly and time-consuming than other methods such as visual inspection. False positives and false negatives can also occur if the sample is not properly prepared or the method is not sensitive enough to detect low levels of contamination. In contrast, these chemical methods are highly sensitive and specific, allowing for accurate detection and quantification of *Alternaria* and patulin in fruit samples (Wang et al., 2016). They are also able to detect low levels of contamination, which is important for ensuring the safety of fruit products for consumption. These methods have been validated and widely used in food safety research and regulatory compliance programs.

### 6.3 Biological method

To reduce the risk of exposure to patulin and *Alternaria* mycotoxin, various biological methods have been developed for these mycotoxin reduction in fruits and their derived products. Some of the established biological methods include; Biocontrol agents (certain microorganisms such as bacteria, yeast, and fungi can inhibit the growth of patulin and *Alternaria* producing fungi by producing certain antagonistic compounds. For instance, the use of bacteria such as Bacillus subtilis and Pseudomonas fluorescens has been found to be effective in reducing patulin levels in apples (Narayanasamy and Narayanasamy, 2013). Similarly, the application of yeast such as Metschnikowia pulcherrima can reduce *Alternaria* mycotoxins in tomatoes.); Plant extracts (certain plant extracts have been found to be effective in reducing the growth of patulin and *Alternaria* producing fungi. For instance, the use of essential oils such as clove and cinnamon oil has been found to be effective in reducing patulin levels in apples (Sivakumar and Bautista-Baños, 2014). Similarly, the use of garlic extract has been found to be effective in reducing *Alternaria* mycotoxins in tomatoes); and Enzymes (some enzymes such as polyphenol oxidase and peroxidase have been reported to reduce patulin levels in fruits (Chen et al., 2023). For example, polyphenol oxidase extracted from apples has been shown to effectively reduce patulin levels in apple juice. Some enzymes such as peroxidase and glucose oxidase have been reported to reduce *Alternaria* mycotoxins in fruits (El Hajj Assaf, 2018). For example, peroxidase extracted from horseradish has been shown to effectively reduce AOH levels in tomatoes (Tittlemier et al., 2020). Glucose oxidase produced by *Aspergillus niger* has been shown to reduce AOH levels in apples (Sun et al., 2023).

It is conspicuous to mention that the biological methods available to detect *Alternaria* and patulin in fruits, require specialized equipment and trained personnel, making them more costly and time-consuming than other methods such as visual inspection. False positives and false negatives can also occur if the sample is not properly prepared or the method is not sensitive enough to detect low levels of contamination. Additionally, the presence of other microorganisms or compounds in the fruit matrix may interfere with the detection of *Alternaria* and patulin (Bartholomew et al., 2021). In contrast, these biological methods are highly sensitive and specific, allowing for accurate detection and quantification of *Alternaria* and patulin in fruit samples. They are also able to detect low levels of contamination, which is important for ensuring the safety of fruit products for consumption (Barkai-Golan and Paster, 2008). These methods have been validated and widely used in food safety research and regulatory compliance programs.

## 7 Gaps and future prospects

There has been significant research on patulin and *Alternaria* toxins in fruit and derived products, there are still several gaps and future prospects for further research to better understand the occurrence, health effects, and control measures of these toxins. While there is a significant amount of research on the occurrence and levels of patulin and *Alternaria* toxins in certain fruits and derived products, such as apple juice and tomato products, there is a lack of information on the occurrence and levels of contamination in other fruits and derived products. For example, more research is needed on the occurrence and levels of patulin and *Alternaria* toxins in tropical fruits, such as mangoes and pineapples, and their derived products. There are inconsistencies in regulations and guidelines for patulin and *Alternaria* toxins in different countries and regions. For example, the European Union has set strict regulations for patulin in apple juice, while the United States has not set any specific regulations for patulin in apple juice. This can create confusion for consumers and manufacturers, and more harmonization of regulations and guidelines is needed. There is a significant amount of research on the health effects and toxicity of patulin, there is limited information on the health effects and toxicity of *Alternaria* toxins. More research is needed to better understand the health effects and toxicity of these toxins, particularly in humans. Analytical methods for the detection and quantification of patulin and *Alternaria* toxins have improved significantly in recent years, but there is still a need for more sensitive and specific methods. This is particularly important for detecting low levels of contamination in fruits and derived products. Moreover, There is a significant amount of research on control measures and mitigation strategies for patulin and *Alternaria* toxins in some fruits and derived products, such as apple juice and tomato products, there is a lack of information on control measures and mitigation strategies for other fruits and derived products. More research is needed to identify effective control measures and mitigation strategies for a wider range of fruits and derived products. There is also a need to investigate emerging mycotoxins in fruits and derived products, which may pose a risk to human health. Some examples of emerging mycotoxins include *beauvericin* and *enniatins*, which are produced by fungi of the genera *Fusarium* and *Aspergillus*.

## 8 Conclusions

It is clear that fungal infections and mycotoxins in fruits and derived products pose a significant risk to human and animal health, making their detection and control crucial. While traditional methods for detecting and removing mycotoxins have been developed, there are still some challenges that need to be addressed, such as the simultaneous detection of multiple mycotoxins and the limitations of physical and chemical methods for mycotoxin removal. Biodegradation has emerged as a potentially viable alternative strategy for mycotoxin control due to its high efficiency, specificity, and lack of pollution. However, further research is needed to understand the complex mechanisms involved in mycotoxin detoxification and to isolate and identify high-purity enzymes for mycotoxin degradation. Advances in genetic engineering may also play a crucial role in this area in the future. Overall, the development of accurate and rapid detection technology and effective mycotoxin control strategies is necessary to ensure the safety of fruits and derived products in human diets.

## Author contributions

SB: Conceptualization, Methodology, Software, Formal analysis, Investigation, Writing - original draft, Data curation, Project admin- tration. JN: Funding acquisition, Conceptualization, Writing - review & editing, Visualization, Project administration. YL: Investigation, Writing - review & editing. GX: Writing - review & editing. SF: Material search and Data collection: LH. All authors contributed to the article and approved the submitted version. 
